# Comparative genome analysis of the immunomodulatory ability of *Lactiplantibacillus plantarum* and *Lactiplantibacillus pentosus* from Japanese pickles

**DOI:** 10.1128/msystems.01575-24

**Published:** 2025-04-29

**Authors:** Yiting Liu, Kazunori Sawada, Takahiko Adachi, Yuta Kino, Tingyu Yin, Naoyuki Yamamoto, Takuji Yamada

**Affiliations:** 1 School of Life Science and Technology, Institute of Science Tokyo, Tokyo, Japan; 2 Innovation Division, Gurunavi, Inc., Tokyo, Japan; 3 Department of Precision Health, Medical Research Institute, The Institute of Medical Engineering, Institute of New Industry Incubation, Institute of Science Tokyo, Tokyo, Japan; 4 Laboratory for Intestinal Microbiota, Juntendo University, Bunkyo City, Tokyo, Japan; 5 Metagen, Inc., Yamagata, Japan; 6 Metagen Theurapeutics, Inc., Yamagata, Japan; 7 digzyme, Inc., Tokyo, Japan; Technion, Haifa, Israel

**Keywords:** fermentation, lactic acid bacteria, comparative genome analysis, immunomodulation, glycerol wall teichoic acid

## Abstract

Lactic acid bacteria (LAB) are pivotal in food preservation and exhibit immunomodulatory effects on interleukin-10 (IL-10) and interleukin-12 (IL-12) production. *Lactiplantibacillus plantarum* (*L. plantarum*) and *Lactiplantibacillus pentosus* (*L. pentosus*) from fermented food are known for their effect; however, a comprehensive comparative genome analysis is needed to identify the linked genes. Here, we investigated the immunomodulatory capability at the genome level of *L. plantarum* and *L. pentosus* strains isolated from Japanese pickles at the genome level, and we further identified their immunomodulation-associated genes using the potential-gene (PG) index derived from the Calinski–Harabasz (CH) index. The results revealed an immunostimulatory clade with strain-specific IL-10 and IL-12 induction and identified key genes via the PG index. Both genes across two species were shown to encode the enzyme *TagF2*, which is crucial for synthesizing poly-glycerol-3-phosphate type wall teichoic acid (poly-GroP WTA), indicating that *TagF2* plays a potential role as an effective microbe-associated-molecular-pattern. *In vivo* analyses confirmed the IL-10-inducing ability of one strain, reinforcing the IL-10-stimulating capacity of its poly-GroP WTA. Subpotential genes in *L. plantarum TagF2-*possessing strains were linked to host‒cell interactions, suggesting that such strains play potential probiotic roles. Collectively, the PG index effectively identified immunomodulation-related genes, thus paving the way for the use of the PG index to detect potential health benefit-associated genes in other LAB species.

**IMPORTANCE:**

Lactic acid bacteria are pivotal in food preservation and exhibit immunomodulatory effects on interleukin-10 (IL-10) and interleukin-12 (IL-12) production. *Lactiplantibacillus plantarum* and *Lactiplantibacillus pentosus* from fermented food are known for such effect, yet comprehensive comparative genome analysis is needed to elucidate the linked genes of the two species. The significance of our research is in observing the immunostimulatory clade with strain-specific cytokine induction and identifying key immunostimulation-related genes encoding enzymes that are crucial for synthesizing a potentially effective microbe-associated-molecular-pattern using the potential-gene index across two species. The further *in vivo* validation reinforced the interleukin-10-stimulating capacity of the identified pattern, and the detected sub-potential genes in *Lactiplantibacillus plantarum* key-gene possessing strains implied the utility of potential-gene index in detecting potential health-benefit-associated genes in other lactic acid bacteria species.

## INTRODUCTION

Lactic acid bacteria (LAB) are Gram-positive, acid-tolerant bacteria (1) that are attracting increasing interest in the food industry because of their metabolic characteristics (2). The fermentation of carbohydrates leads to the production of lactic acid and various beneficial metabolites that contribute to aroma, flavor, health effects, and shelf life in a wide range of food products (3–5). Hence, many LAB species have a long history of human use (6) and have been deemed as “generally recognized as safe” or received a “qualified presumption of safety” by the Food and Drug Administration and the European Food Safety Authority, respectively (7). Certain LAB strains are marketed as probiotics, offering health benefits beyond basic nutrition, including gastrointestinal disorder management, immunomodulation, intestinal microbiome balance maintenance, and pathogens prevention (8–12). Furthermore, the use of food-grade LAB as vectors for vaccine (13) and therapeutic delivery (14) is an active area of research. Nevertheless, the diverse and profound health-benefit potential of LAB, particularly their immunoregulatory capabilities, underscores the need for further genome-level exploration to advance their bioengineering application.

LAB regulate the host immune system via interactions with the gut immune cells and potently induce the expression of both anti- and pro-inflammatory cytokines, including interleukin-10 (IL-10) and interleukin-12 (IL-12) in the host gut (15, 16). Studies have elucidated the signalling pathways underlying these host–bacteria interactions, highlighting the role of innate immune pattern recognition. These pathways involve microbe-associated molecular patterns (MAMPs) of LAB and pattern recognition receptors (PRRs) on immune cells, such as toll-like receptors (TLRs) and nod-like receptors, which produce respective cytokines via the early- and late-phase activation of the transcription factor nuclear factor-kappa B (NF-κB) and mitogen-activated protein kinases (MAPKs) (17–19). Effective MAMPs from LAB were reported, including lipopolysaccharides, lipoproteins, polysaccharide A, peptidoglycans, lipoteichoic acid (LTA), and microbial RNA and DNA containing CpG motifs (20). Crosstalk between these MAMPs and PRRs subsequently induces diverse immune responses in a strain-dependent manner (21, 22). For instance, lipoproteins from *Lactiplantibacillus plantarum* (*L. plantarum*) WCFS1 contribute to the activation of TLR2 signalling and downstream anti-inflammatory responses. Moreover, TLR3-mediated immune responses associated with LTA have been observed in other strains of *L. plantarum* (23). CpG oligodeoxynucleotides enhance pro-inflammatory IL-12 and tumor necrosis factor alpha production by modulating TLR9 expression, which in turn inhibits MAPKs and NF-κB signalling (24). Despite significant advances in understanding LAB-immune cell interactions, the key genes associated with MAMPs that represent strain-level individuality in cytokine induction remain unexplored.

With the advent of next-generation high-throughput sequencing, genome-level exploration has provided insights into genetic variances in niche adaptation, lifestyle differences, and future development for industrial fermentation using LAB (25, 26). An examination of 54 *L*. *plantarum* genomes revealed the nomadic characteristics of this bacterium, indicating that *L. plantarum* independently acquired and retained the functions from the habitat (27). A comparison of *L. plantarum* genomes unveiled the functionalities of beneficial organic acid biosynthesis genes, highlighting their potential for large-scale fermentation (28, 29). *Lactiplantibacillus pentosus* (*L. pentosus*), which is taxonomically close to *L. plantarum*, has been recognized for its safety, functionality, and probiotic potential related to exopolysaccharide biosynthesis (30–32). Despite considerable progress in exploring the *L. plantarum* and *L. pentosus* genomes, significant questions remain concerning the genetic differences in the immunomodulatory capabilities of *L. plantarum* and *L. pentosus* strains. There is also a critical need for an innovative comparative genome analysis approach to identify immunostimulation-related genes with IL-10- and IL-12-inducing activities.

Building on previous reports about the immunostimulatory effects of *L. plantarum* and *L. pentosus* from various fermented foods (33–35), pickles are good resources for the isolation of these two species because of their rich plant-originated nutrients. We aimed to determine whether strains originating from Japanese pickles are effectively immunomodulatory or not. Given the established antagonistic role of IL-10 and IL-12 in modulating immune responses (36, 37), these strains might exclusively stimulate the production of either IL-10 or IL-12; this specificity could suggest their potential application in restoring immune homeostasis. We examined the genomic relationships with the constructed pangenomes of the strains. IL-10 and IL-12 levels stimulated by 61 LAB strains were measured, and the strains were subsequently categorized into different groups on the basis of the measurements. The potential-gene (PG) index pinpointed vital and subpotential immunomodulation-related genes in two species. The IL-10-inducing ability of one vital gene-possessing strain was confirmed via *in vivo* validation in a mouse model.

## RESULTS

### Phylogenetically close LAB from Japanese pickles showed cytokine-inducing proximity in clade

**Fig 1:**
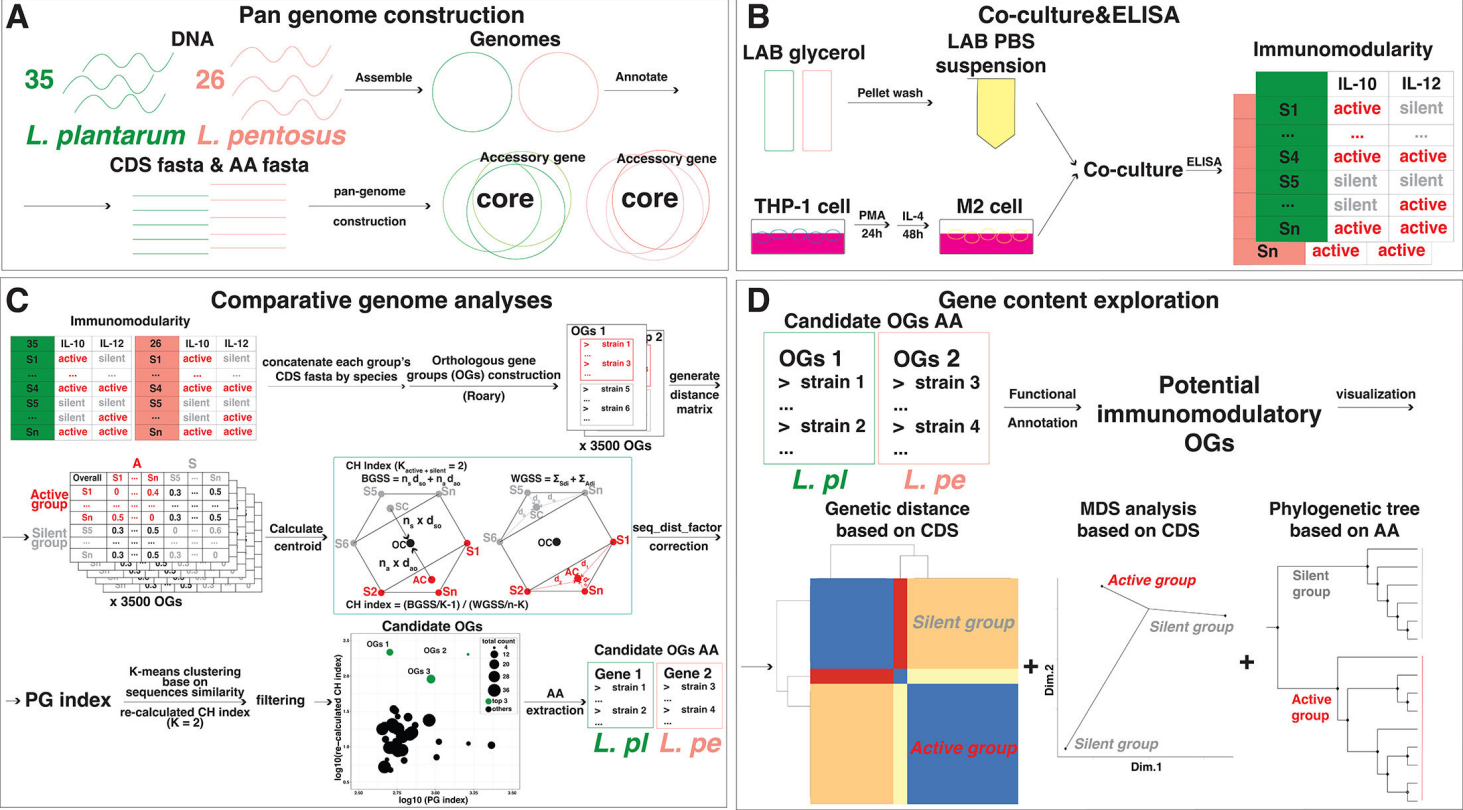
Overview of the analysis pipeline. (**A**) Genome assembly, annotation, and pangenome construction of local strains across *L. plantarum* (*L. pl*) and *L. pentosus* (*L. pe*); (**B**) the immunomodulation assessment of *L. plantarum* and *L. pentosus*, respectively; (**C**) detection of candidate OGs via comparative genome analysis with PG index; (**D**) *in silico* gene content exploration and genetic distance visualization of potential immunomodulation-related genes. Strains of *L. plantarum* are represented in green, and strains of *L. pentosus* are represented in pink. CDS, coding sequence; AA, amino acid; PBS, phosphate-buffered saline; sus, suspension; CH, Calinski–Harabasz; S, strain; AC, active group distance matrix centroid; SC, silent group distance matrix centroid; OC, overall distance matrix centroid.

By assessing the IL-10 and IL-12 inducing capacity and analyzing the genomes of 61 isolated strains and two type strains across *L. plantarum* and *L. pentosus*, we aimed to determine their immunomodulatory proximity at the genome level (Fig. 1A and B). The taxonomy of these strains was reconfirmed, and we observed high average nucleotide identity and tetranucleotide frequency values within the two species (Fig. S1 and S2). Single-nucleotide polymorphism (SNP) phylogenetic information combined with the genomic statistics of the two species demonstrated differences from the intra-clade comparison (Fig. S3), whereas the orthologous gene groups (OGs) content of each strain slightly differed across two species (Fig. S4). In addition, compared with isolated strains of *L. plantarum*, isolated strains of *L. pentosus* generally exhibited larger genome sizes and higher GC contents (Fig. S3B). This phylogenetic proximity of the genomic statistics shown in the clades led us to postulate that such a pattern would manifest as immunomodulation by these isolated strains.

**Fig 2:**
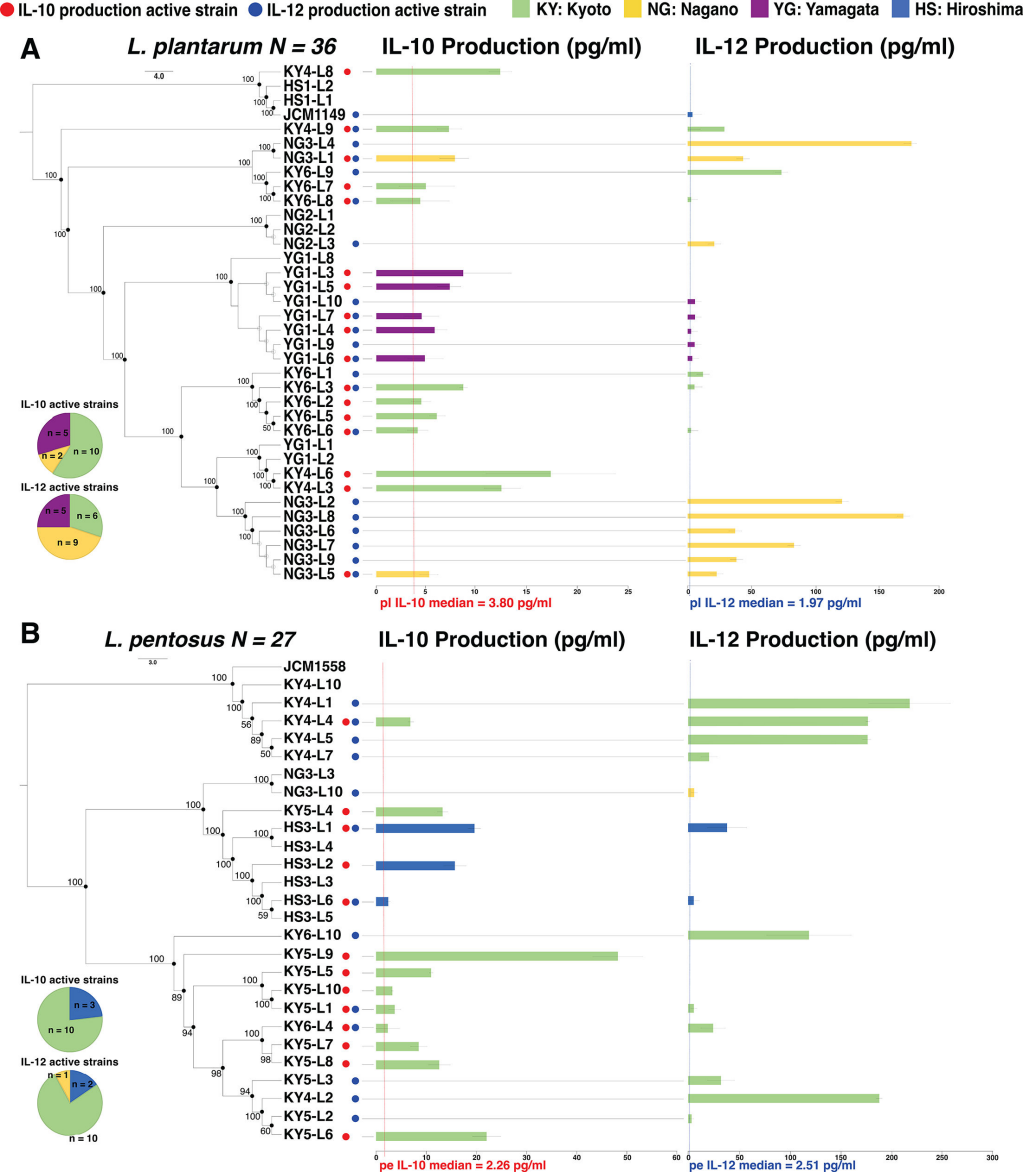
The immunostimulatory activities of IL-10 and IL-12 across *L. plantarum* and *L. pentosus*. (**A**) Immunostimulatory clades varied in *L. plantarum* (*N* = 36, median = 3.80 pg/mL); (**B**) immunostimulatory clades varied in *L. pentosus* (*N* = 27, median = 2.26 pg/mL). The immunostimulatory variances across clades were shown by the combination of an SNP phylogenetic tree and ELISA measurements of IL-10 and IL-12 levels. Highly supported nodes are indicated with a closed circle and actual value (bootstrap support value ≥50%). Active strains were defined as those whose concentrations were above the median. Red and blue points indicated the IL-10- and IL-12-stimulating active strains, respectively. Square symbols of different colors represent different prefectures. The pie charts showed the number of IL-10- and IL-12-active strains from different prefectures.

The investigation of cytokine induction revealed that the average production of IL-12 was significantly greater than that of IL-10 in both species (Table S1). Enzyme-linked immunosorbent assay (ELISA) measurements and the SNP phylogenetic trees further showed the immunoregulatory clades across the two species (Fig. 2). Clades presenting different levels of cytokine inducibility were observed in *L. plantarum* (Fig. 2A; Table S2). The strongest IL-12-inducing clade was composed of strains from NG3-L2 to NG3-L5, exhibiting considerable variability. IL-10 stimulation clades were shown on two branches. One branch elicited the highest average level of IL-10 induction and included strains KY4-L3 to YG1-L2; however, neither YG1-L1 nor YG1-L2 showed IL-10 or IL-12 inducibility. Another branch included strains KY6-L1 to KY6-L6, which induced comparatively lower and similar IL-10 production. Interestingly, other YG-originated strains displayed a conservative genomic manner in a weak IL-10-stimulating clade, with five strains lacking IL-10 stimulatory activity. Clades with opposite cytokine-inducing abilities were also found in *L. pentosus* (Fig. 2B; Table S3). The IL-10-inducing clade was composed of KY6-L10 to KY5-L6 with diverse elicited IL-10 levels, whereas four strains of this clade didn’t stimulate IL-10 production. IL-12-modulating clade, including JCM1558 to KY4-L7, displayed various IL-12 production, although KY4-L7 induced a low level of IL-12 compared to the other active strains in this clade. Notably, most strains of either the IL-10-stimulating or the IL-12-inducing clades enhanced sole cytokine production with strain individuality across two species.

### Shared immunomodulation-related genes were observed across cytokine-inducing clades

The classification of strains as active or silent is detailed in the Materials and Methods. The identification of immunostimulatory clades spanning *L. plantarum* and *L. pentosus* suggested that these phylogenetically close active strains might possess the common immunostimulation-related genes. We hypothesized that these genes would exhibit high intra-cluster genetic similarity within either active or silent groups. Conversely, the inter-cluster genetic distance of these genes should be large, reflecting substantial dissimilarity between the active and silent groups. Such a genetic distance pattern would result in high scores using evaluating metrics for clustering analysis. This hypothesis led us to identify the common genes by employing an evaluation metric for comparative genome analysis. Thus, we applied the PG index for the detection (Fig. 1C).

**Fig 3:**
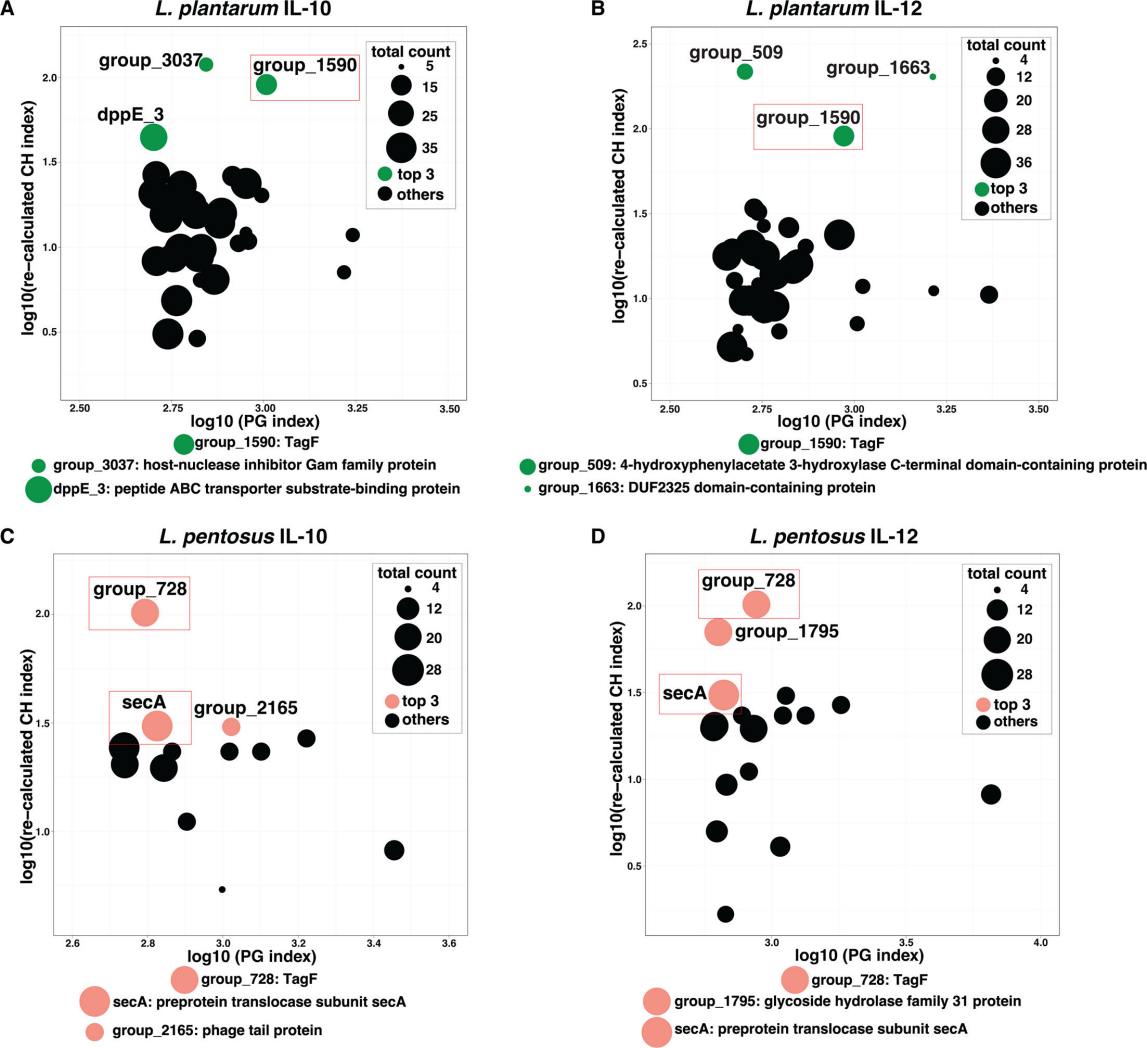
The top 2% of orthologous gene groups screened out by PG index and re-calculated CH index (K-cluster = 2). (A and B) The top 2% orthologous gene groups screened out from IL-10 and IL-12 comparison analysis for *L. plantarum*; (C and D) the top 2% orthologous gene groups screened out from IL-10 and IL-12 for *L. pentosus*. *P* < 2.2e–16 (Kruskal‒Wallis rank sum test). The top three orthologous gene groups ranked by re-calculated CH index are shown in green (*L. plantarum*) and pink (*L. pentosus*) color. Those overlaps in both IL-10 and IL-12 comparative analyses are highlighted with red rectangles. The total count represents the number of strains contained in each orthologous gene group. The orthologous gene groups with a re-calculated CH index of 0 were not plotted.

In each qualified OG screened out by the PG index, genes of corresponding immunomodulatory (active and silent) groups might show parallel genetic disparity between the two groups. Therefore, we designed the screening process for the immunomodulation-related genes using the PG index and recalculated the Calinski‒Harabasz (CH) index (see Materials and Methods). Since both IL-10 and IL-12 are cytokines induced by LAB strains, we hypothesized that there might be genetic similarities in the MAMPs of those LAB strains. Therefore, we focused on the overlaps from the screening analysis of the IL-10 and IL-12 comparison (Fig. 3; Tables S6 to S9). Among the isolated *L. plantarum* strains, the OG for *TagF* (*group_1590*) consistently ranked among the top three, with a high recalculated CH index by genetic distance (K = 2), emerging as significant in both the IL-10 and the IL-12 comparisons (Fig. 3A and B; Tables S6 and S7). Two shared gene groups, *TagF* (*group_728*) and secA, were identified with high recalculated CH indices across isolated *L. pentosus* strains from the IL-10 and IL-12 comparisons, indicating distinct genetic distances between the active and silent groups (Fig. 3C and D; Tables S8 and S9).

### Putative MAMP biosynthetic genes were identified with opposite cytokine-inducing capacities

**Fig 4:**
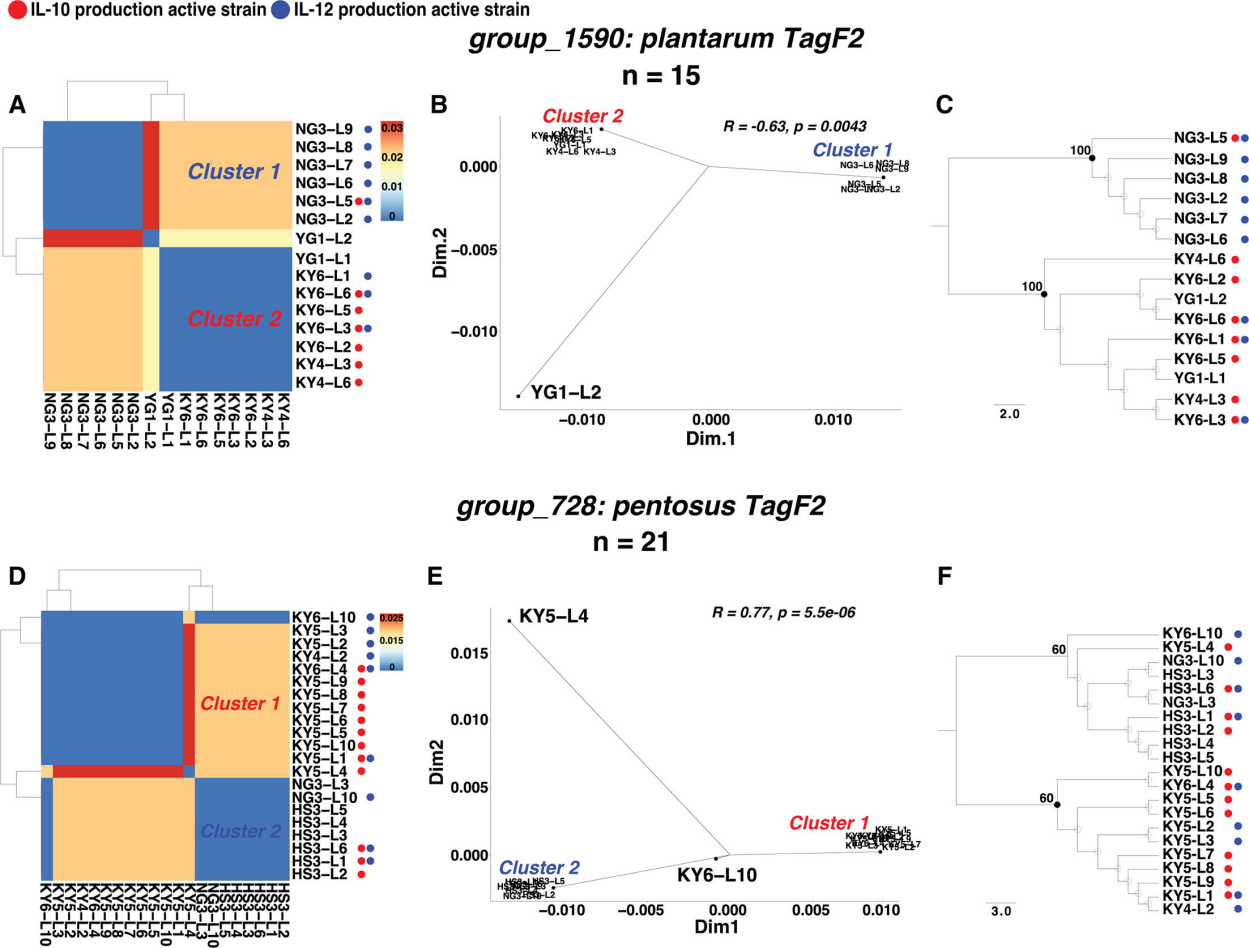
Genetic distance analysis of potential immunomodulation-related genes. (**A** and **B**) Genetic distance of *TagF2* (*group_1590*) shown in heatmaps and MDS analysis plot (*n* = 15); (**D** and **E**) genetic distance of *TagF2* (*group_728*) shown in heatmaps and MDS analysis plot (*n* = 21). (**C** and **F**) phylogenetic trees of *TagF2* (*group_1590*) and *TagF2* (*group_728*) based on their amino acid sequences, respectively. The number of strains that harbor the corresponding potential genes is indicated in n. Highly supported nodes are indicated with a closed circle and actual value (bootstrap support value ≥50%). Red points indicate the IL-10-stimulating active strains, and blue points indicate the IL-12-stimulating active strains. Clusters are shown in red or blue color to represent IL-10 or IL-12 activity.

**Fig 5:**
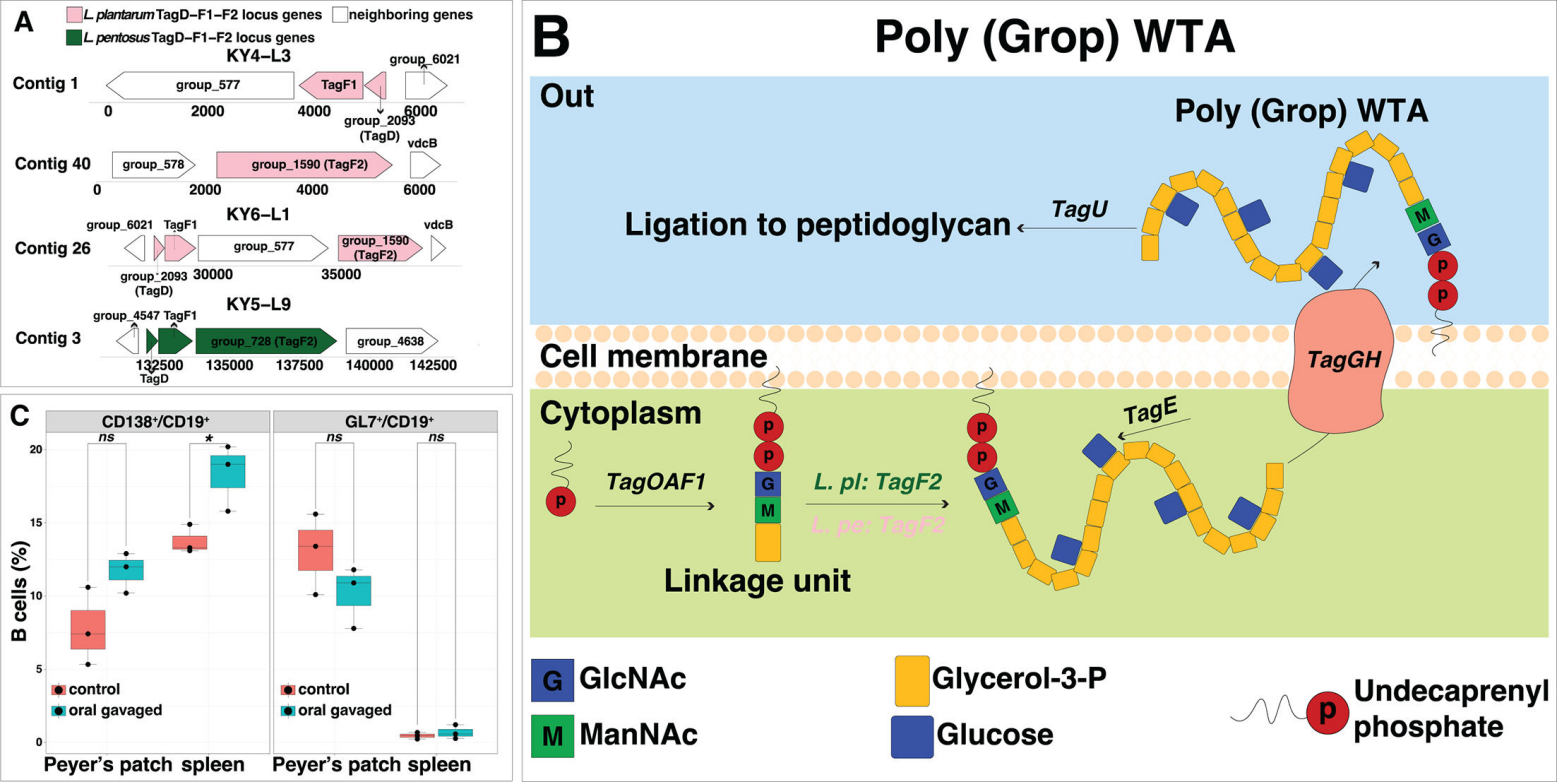
Putative genes for schematic MAMP biosynthesis in *TagF2* possessing strains across two species. (**A**) *TagD-F1-F2* locus found in KY4-L3 and KY5-L9, and the same locus conserved by other *TagF2* (*group_1590*) and *TagF2* (*group_728*) possessing strains were shown in Fig. S6 and S7; (B) schematic biosynthesis pathway of poly-GroP WTA in *TagF2* possessing strains across *L. plantarum* and *L. pentosus*; (**C**) the proportion of plasmablast (CD138^+^/CD19^+^) and germinal center (GL7^+^/CD19^+^) B cells from Peyer’s patches and spleens in the control and KY4- L3 orally gavaged mouse groups, as measured by flow cytometry. *P*-value was calculated using one-way analysis of variance followed by Bonferroni correction (*, *P*-value < 0.05; ns, non-significant). *L. pl, L. plantarum*; *L. pe, L. pentosus*; *Group_577*, histidine kinase; *Group_578*, Wxl domain protein; vdcB, UbiX-like flavin prenyltransferase; *Group_6021*, hypothetical protein; *Group_4547*, hypothetical protein; *Group_4838*, the CDP-glycerol glycerophosphotransferase.

We identified candidate genes for immunostimulation from *L. plantarum* and *L. pentosus* encoding *TagF*, a GroP polymerase. This gene is involved in the biosynthesis of poly-glycerol-3-phosphate type wall teichoic acid (poly-GroP WTA), a potential MAMP for cytokine induction (38, 39). Multidimensional scaling analysis (MDS) and phylogenetic analysis confirmed genetic distances between active and silent groups within candidate genes (Fig. 4B, C, E, and F). Therefore, we defined these genes as potential immunomodulation-related genes in the two species. We named those separated clusters as “cluster 1” and “cluster 2” among the candidate genes. With respect to the genetic distances of the candidate OG in *L. plantarum* (*group_1590*), cluster 2 encompassing strains of IL-10-stimulating clade was distant from cluster 1 represented by IL-12-inducing clade (Fig. 4A through C). Similarly, the genetic distances of the candidate OG in *L. pentosus* (*group_728*) showed that cluster 1, which comprised the IL-10-modulating clade, was distant from cluster 2 of strains that could barely stimulate IL-10 or IL-12 production (Fig. 4D through F). The *TagD1-TagF1-TagF2* (*tag-locus*) gene cluster in *L. plantarum* WCFS1 plays a pivotal role in the production of poly-GroP WTA, and the size differences between encoded *TagF1* and *TagF2*-implicated *TagF1* might act as the GroP primase (*TagB*) (38). Additional *TagF* genes were found in *group_1590*- and *group_728*-conserving strains, noting distinct length differences in their amino acid sequences. The *TagF* proteins encoded by *group_1590* and *group_728* were longer, leading us to postulate that the shorter variants function as *TagF1* (GroP primase), whereas the longer one represents *TagF2*. Hence, this classification revealed similar gene clusters across immunomodulation-related genes conserving strains in the two species (Fig. 5A; Fig. S6 and S7).

In *L. plantarum*, KY4-L3 and KY4-L6 conserved a sequential gene arrangement of *TagD* (*group_2093*)*-TagF1* and *TagF2* (*group_1590*) in their genomes, while the KY6 strains harboured *tag-locus* sequentially (Fig. 5A; Fig. S6A). YG-L1 within cluster 2 of *TagF2* (*group_1590*) exhibited the same gene locus arrangement, highlighting the potential of YG1-L1 for synthesizing poly-GroP WTA. *TagF2* proteins encoded by *group_1590* in cluster 1 contained 1,092 residues, while *TagF2* in cluster 2 comprised a shorter amino acid sequence of 665 residues. Further examination revealed that the neighboring gene (*group_577*) in strains KY4-L3, KY4-L6, and YG2-L2 likely functions as a histidine kinase involved in signal transduction across the cellular membrane, whereas the neighboring gene (*group_578*) in the KY6 strains aligned with the WxL domain protein (Fig. S6A). Strains in Cluster 1 presented sequentially arranged *TagD* (*group_2093*)*-TagF1* (Fig. S6B). Various genes (*group_1591*, *group_1592*, *group_1593,* and *group_1594*) neighboring *TagF2* (*group_1590*) of strains in cluster 1 were aligned with CDP-glycerol glycerophosphotransferase. Interestingly, their size (386 residues) was slightly similar to that of *TagF1* (399 residues).

A similar *tag-locus* was observed in the isolated *L. pentosus* strains (Fig. 5A and Fig. S7). The *TagD-TagF1-TagF2* (*group_728*) was consistently conserved across isolated strains, except for the OG (*group_729*) found in strain KY6-L10, which was situated between *TagF1* and *TagF2* (*group_728*). The size difference between *TagF1* (405 residues) and *TagF2* (*group_728*: 1675 residues) sequences indicated that *TagF1* might function as GroP primase (*TagB*). Interestingly, the *TagF2* proteins encoded by *group_728* in strain KY6-L10 comprised a shorter length with 905 residues, and the IL-12-inducing profile of this strain aligned with the *TagF2* (*group_728*) length variances compared to other phylogenetically close strains (Fig. 2B and 4D). Further exploration of another copy of *TagF2* (*group_4638*) showed the size was 1,092 residues, smaller than that of *TagF2* (*group_728*). The observed genetic distances and variations in the size of the encoded enzyme indicated differences in poly-GroP WTA synthesized by the candidate genes of the respective clusters, which might lead to differences in cytokine induction. The other WTA biosynthesis-related genes were distributed across *TagF2* (*group_1590*) and *TagF2* (*group_728*) conserving strains, further pinpointing their potential for poly-GroP WTA biosynthesis and therefore suggesting the cytokine-inducing capacity of this cell wall component (Fig. S8 and S9).

Since the IL-10-inducing strain KY4-L3 displayed the potentiality of poly-Grop WTA biosynthesis, we selected it for *in vivo* validation on the mouse model (Fig. 5C). IL-10 can interrupt the proliferation of B cells in the germinal center via promotion of plasmablast differentiation (40, 41). With the oral gavage of KY4-L3 to the model mice, the number of CD138^+^/CD19^+^ plasmablasts significantly increased in the spleen and showed an increasing trend in Peyer’s patch. Additionally, the number of GL7^+^/CD19^+^ presenting germinal center B cells demonstrated tended to decrease in Peyer’s patch. These observations suggested the potential IL-10 inducibility of KY4-L3, further implying that the poly-GroP WTA synthesized by KY4-L3 could serve as an effective MAMP for activation of anti-inflammatory cytokine production (Fig. 5B). Furthermore, we identified the subpotential immunostimulatory genes based only on the PG index. The function exploration of OGs with the top two PG indices across *L. plantarum* and *L. pentosus* identified two subpotential candidate genes, which were aligned to the WxL domain (*group_511*) and LPXTG-motif cell wall anchor proteins (*group_3159*) (Fig. S5). The WxL domain and LPXTG-motif cell wall anchor proteins are associated with facilitating cellular communication or adhesion to human gut epithelial cells (42–44). The subpotential candidate genes conserving strains overlapped with some of the *TagF2* (group_1590) conserving strains, which highlighted their potential probiotic role as commensal bacteria within the human gut (Fig. 4A; Fig. S5).

## DISCUSSION

The immunomodulatory clades with strain-dependent IL-10 or IL-12 induction across *L. plantarum* and *L. pentosus* were discovered. Either IL-10-inducing or IL-12-stimulating clades of the two species displayed sole capacity, which suggested their ability to stimulate other anti- and pro-inflammatory cytokines. Furthermore, the exclusivity of cytokine production provided by isolated strains also elucidated an insight for the co-application of opposite cytokine-inducing strains for restoring immune homeostasis (45–47). We have detected immunomodulation-active strains with cytokine-stimulating individuality from Japanese pickles, and such strain-specific manner is consistent to the previous reports (21, 22). The distinct profiles of clades in isolated *L. plantarum* strains revealed a spectrum of activities; intriguingly, three strains demonstrated dual immunoregulatory effects (Table S2). The *L. pentosus* cytokines-stimulating strains also showed immunomodulation individuality, in which two strains enhanced both IL-10 and IL-12 production (Table S3). In addition, the average IL-10 and IL-12 production varied across prefectures in the two species (Table S4 and S5).

The mobile gene elements (plasmids, transposons, and bacteriophages), adaptive immune systems genes (CRISPR-Cas elements and bacteriocins), genes responsible for stress resistance, bile salt hydrolase activity, adhesion ability, and gut persistence were characterized and identified as probiotic marker genes, and 70% of the probiotic marker genes were determined to be the core/soft-core genomes (95% ≤ strains ≤ 100%) via pangenome analysis (48). The probiotic marker genes were identified using a conservative threshold here: an OG was considered significant if present in ≥80% of the active strains, while ≤50% of the silent strains were within their respective IL-10- and IL-12-inducing groups (Fig. S10; Tables S10 to S13). These probiotic marker genes were predominantly annotated as phage-related proteins, transcriptional regulators, and cell adhesion proteins, indicating their role in mobile genetic elements and cell-to-cell interactions. While these probiotic marker genes only suggested the cytokine-inducing capabilities in active strains, this traditional analysis failed to detect genes directly involved in effective MAMPs. Intriguingly, the PG index determined *TagF2* as the key immunoregulation-associated gene involved in the biosynthesis of poly-Grop WTA, implying the effective MAMPs role of this cell wall component in cytokines induction. The subsequent exploration of the *TagD-TagF1-TagF2* locus in two species appears to confirm such a possibility. Notably, this *tag-locus* in *L. pentosus* hasn’t been reported in the context of cytokine inductions. Nevertheless, the ability to biosynthesize poly-GroP WTA conserved in these potential WTA-producing strains can be attained by the absence of *TagH* and *TagB*. The glycerol WTA was not detected effectively stimulating the immune cell to produce cytokines via TLR2/6-mediated NF-κB activation signaling pathway compared to LTA. Additionally, the purified poly-GroP WTA was not directly involved in immunomodulation, but the appropriately isolated glycerol WTA was observed to enhance the induction of IL-12/p70 (38). Similarly, the high IL-12-inducing activities of *TagF2* (*group_1590*) conserving cluster expressed such cytokine-inducing characteristics, further emphasizing the impact of poly-Grop WTA on stimulating the specific cytokine production (Fig. 2A and 3A). *In vivo* validation confirmed the IL-10 inducibility of one *TagF2* (*group_1590*) possessing *L. plantarum*, shedding light on the anti-inflammatory cytokines inducing ability of poly-GroP WTA produced by the IL-10-inducing *TagF2* (*group_1590*) cluster. In addition, the amino acids and size differences between IL-10- and IL-12-stimulating clusters across two species suggested the differences in synthesized poly-GroP WTA, which expressed different effects on cytokine induction. Indeed, silent strain (YG1-L1) was grouped within poly-GroP WTA-producing cluster 2 (Fig. 4A), and such a genetic relationship implied its bioengineering potential for future probiotic development. Unfortunately, the poly-GroP WTA from *TagF2* (*group_1590* and *group_728*) was not extracted, which could undermine the potential MAMP role of this cell envelope component.

Our research has revealed the immunostimulatory relationship of isolated strains, as particularly evident in the case of the sole cytokine inducibility. The detection of *TagF2* in two species led us to identify the putative immunoregulatory impact of poly-GroP WTA, implying that they might have interacted directly or been involved as a supporter via cell PRRs. Although the direct immunomodulatory effect of poly-GroP WTA is absent here, the mouse model indicated the anti-inflammatory cytokine-stimulating role of this cell wall component. By utilizing the introduced PG index-based gene screening analysis, the comparative genome analysis pipeline effectively identified immunomodulation-linked genes in two species, offering valuable insights for future application of this index to discover health benefit-related genes of potential probiotic LAB.

## MATERIALS AND METHODS

### Sample collection and sequencing

Two type strains were obtained from the Japan Collection of Microorganisms (JCM). The original strains were collected from Japanese pickles (*N* = 61). These Japanese pickles were provided by eight manufacturers from four prefectures: Hiroshima (HS), Kyoto (KY), Nagano (NG), and Yamagata (YG). The numbers after each prefecture represent different manufacturers. The isolation protocol for the strains was as follows. *L. plantarum* selective medium (LPSM) selective agar medium (49) containing 4 mg/L of ciprofloxacin was used for LAB isolation. The LPSM was modified as follows. Instead of bromocresol purple, calcium carbonate (10 g/L) was added to autoclaved medium after separate sterilization at 180°C for 1 h. The washed solution (sterile saline) of Japanese pickles was plated on LPSM agar medium and anaerobically incubated at 30°C or 37°C for more than 24 h. Colonies surrounded by clear zones were picked as candidates. The selected candidates were identified as *L. plantarum* via PCR amplification of the 16S rRNA gene with the primers IDL04F and IDL62R (50). Positive colonies were purified by single-colony isolation, reconfirmed via 16S rRNA gene PCR amplification, and subsequently stored in Man–Rogosa–Sharpe (MRS) medium supplemented with 10% glycerol at −80°C. For DNA extraction, bacterial cells were prepared by culturing candidate strains in MRS medium broth (Becton, Dickinson and Company, Franklin Lakes, NJ, USA) and incubated at 30°C or 37°C for 24 h and centrifuging the culture broth at 20,400 × *g*, 4°C for 5 min. DNA was extracted using an ISOPLANT solution kit (Nippon Gene, Tokyo, Japan), and the extracted DNA was dissolved in Tris-EDTA buffer and purified with Genomic DNA Clean & Concentrator kit-10 (Zymo Research, Irvine, CA, USA) according to the manufacturer’s instructions. Purification was prepared for the library using the TruSeq DNA PCR-Free kit (Illumina, Inc., San Diego, CA, USA) following the manufacturer’s protocols, and sequencing was performed on the HiSeq X platform to generate 150 bp paired-end reads.

### Genome curation

Genomes were curated as follows (Fig. 1A). Quality control of the reads was confirmed using fastQC (version 0.12.1; default) (51). The raw reads were trimmed by fastp (version 0.23.4; option: -q 20 -L 20 -w 8), and they were assembled into draft genomes using SPAdes (version 3.15.5; optional kmers: -k 21,33,55,77,99,127) (52, 53). Genome completeness and contamination were calculated by CheckM (version 1.2.2; default) (54), and genomes with contamination over 5% were re-assembled using UniCycler (version 0.5.0; default) (55). Genome size and GC content were computed using QUAST (version 5.2.0; default) (56). Genomes were annotated by Prokka (version 1.14.6; default), and OGs and pangenomes were constructed using Roary (version 3.13.0; default) (57, 58). The SNP genes were extracted from core-gene alignment and trimmed using snp-sites (version 2.5.1, default) and trimAl (version v1.4.rev15; option: -phylip -gappyout) (59, 60). The SNP phylogenetic trees were constructed using RAxML (version 8.2.12; option: -f a -x 12345 -p 12345 -# 100 -m GTRGAMMA) (61). Visualization of the genome statistics and OG content was performed using ggtree (version 3.8.2), ComplexHeatmap (version 2.16.0), and ggplot2 (version 3.4.4) (62–65).

The amino acid (AA) sequences of housekeeping genes *mutL*, *pheS,* and *recA* were extracted from isolated and reference strains. These sequences were concatenated and integrated using the in-house script. The sequences were aligned and trimmed using Clustal Omega (version 1.2.4; default) (66) and trimAl (version 1.4. rev15; option: -phylip -keepseqs). The phylogenetic trees based on housekeeping genes were estimated using RAxML (version 8.2.12; option: -f a -x 12345 -p 12345 -# 100 -m PROTGAMMAWAG). Thirty-five isolated strains re-confirmed as *L. plantarum* and 26 isolated strains confirmed as *L. pentosus* were chosen for subsequent analysis. The pairwise average nucleotide and tetranucleotide frequency values were calculated using the average_nucleotide_identity.py script from the pyani package (version v0.2.12; option: -m ANIb -g) (67).

### Immunomodulatory analysis of isolated and type strains

Immunomodulatory analysis of IL-10 and IL-12 induction was performed (Fig. 1B). Glycerol stocks of the isolated and type strain glycerol stocks were grown anaerobically on MRS agar media for 18 h at 30°C. Colonies were randomly selected and inoculated into MRS broth for 24 h at 30°C as a preculture. The optical density of bacterial MRS broth was measured at 600 nm using BioPhotometer Plus (Eppendorf), and the precultures were adjusted to an optical density of 0.1 for the main cultures. The corresponding volume of precultures was inoculated into MRS broth for 24 h at 30°C. A volume of 1 mL of each main culture was transferred to a sterilized tube and centrifuged at 20,400 × *g*, 4°C for 10 min, after which the bacterial pellet was washed with 0.85% NaCl solution. The process was repeated, and the supernatant was discarded. Each pellet was weighed and adjusted to 10 mg for the preparation of the phosphate-buffered saline (PBS) suspension. A corresponding volume from each main culture was transferred to a sterilized tube and heat-treated at 80°C for 30 min. The dead bacterial cells were harvested via centrifugation at 20,400 × *g*, 20°C for 10 min and subsequently washed with 1× PBS. This process was repeated to ensure thorough washing. The washed bacterial cells were suspended in 1× PBS and stored at −20°C. The 1× PBS was prepared from 10× PBS (Gibco).

M2 macrophage culture and ELISA measurements were performed. Human THP-1 monocyte cells were obtained from the RIKEN Cell Bank (JRCB0112). M2 macrophage cultures were prepared in triplicate for each LAB strain. THP-1 monocytes were cultured in Roswell Park Memorial Institute medium (RPMI 1640) supplemented with 10% FBS, 100 U/mL penicillin, and 100 µg/mL streptomycin at 37°C in a 5% CO_2_ incubator. THP-1 monocytes were incubated with 100 nM phorbol 2-myristate 13-acetate (PMA; Adipogen Life Science, Liestal, Switzerland) at 5 × 10^5^ cells per well in a 48-well culture plate for 24 h to differentiate into M0 macrophages. Macrophage M2 macrophage polarization was achieved by incubating M0 macrophages with 20 ng/mL interleukin-4 (Peprotech, Cranbury, NJ, USA) for 24 h. Bacterial cell concentrations of LAB in PBS suspensions were determined using a cell imager (Bio-Rad). Volumes were calculated to achieve a seeding density of 50 × 5 × 10^5^ cells per well. Each suspension was added to wells with M2 macrophages for 24 h co-culture at 37°C under 5% CO_2_. Supernatants from co-cultures were collected. IL-10 and IL-12 levels were measured using IL-10 (BioLegend, San Diego, CA, USA) and IL-12 p40 (R&D Systems, Minneapolis, MN, USA) ELISA kits following the manufacturer’s instructions. Fluorescence was measured in duplicate using a Varioskan LUX plate reader (Thermo Fisher Scientific, Waltham, MA, USA). IL-10 and IL-12 production by each strain was quantified from the mean value of the duplicate measurements. Strains were classified as “active” if their cytokine production was greater than or equal to the median value of all strains tested; those with cytokine production below the median were classified as “silent.” The strains were classified into four groups: “IL-10 active,” “IL-10 silent,” “IL-12 active,” and “IL-12 silent.”

### Comparative genome analysis between the active and silent groups

OGs were constructed from 61 LAB strains using Roary. OGs represented in only a single strain or absent were excluded. Only OGs conserved across multiple strains within active or silent groups were retained for further comparative genome analysis (Fig. 1C).

The CH index is conventionally employed to gauge the quality of clustering and to determine the optimal number of clusters within a data set (68). We adapted the CH index for each OG between the active and silent strain groups and introduced a sequence distribution factor (seq_dist_factor) to adjust it to the PG index, which prioritized genes potentially contributing to immunomodulatory activity related to IL-10 and IL-12 production. The PG index was calculated as follows.

Three matrices for each OG were calculated for index calculation: one for the active group, one for the silent group, and one for the overall matrix of the combined active and silent groups. The between-group sum of squares (BGSS) was calculated using the centroids of the active group, silent group, and overall matrix as follows:


$$BGSS=\sum_{i=1}^{k}n_{i}∥C_{i}-C{∥}^{2},$$

where *n_i_* is the number of strains in each group, *c_i_* is the centroid of the active or silent group, and *c* is the centroid of the overall matrix. BGSS was computed by measuring the distances between the active and overall centroids, and the silent and overall centroids and then aggregating those values to derive the final BGSS.

The within-group sum of squares (WGSS) was calculated as follows:


$$WGSS=\sum_{i=1X\in C_{i}}∥X-C_{i}{∥}^{2},$$

where *X* represents strains in their respective group, and *c_i_* is the group centroid. WGSS was determined by measuring the distances between each strain and its corresponding group centroid and summing those values to obtain the final WGSS.

The CH index was the ratio of BGSS to WGSS, normalized by their respective degrees of freedom. The seq_dist_factor was multiplied by the CH index, forming the PG index as follows:


$$CH\;index=\frac{BGSS/\left(K-1\right)}{WGSS/\left(n-K\right)},$$

$$seq\_dist\_factor={\frac{N_{overall}}{zero\_value\_count}}_{overall}$$

PGindex=CHindex×seq_dist_factor,

where *K* is fixed to two clusters (active and silent), *N*_overall_ is the number of strains containing the OG, and zero_value_count_overall_ is the count of zero values in the overall distance matrix.

The top 2% of OGs were selected based on the PG index. To screen out the qualified OG candidates for immunomodulatory activity, k-means clustering analysis based on sequence similarity and then the CH index recalculation were applied. The recalculated CH indices (in the case of K = 2) were ranked in descending order, and the top three OGs that overlapped in both the IL-10 and the IL-12 comparisons across two species were retained for further gene function exploration. The significant differences in the PG index across active and silent groups were determined by the Kruskal‒Wallis test, and the significance was confirmed by a pairwise Wilcoxon test with Bonferroni correction (*P* < 0.05).

### Gene content exploration

AA sequences for each candidate gene were extracted using the in-house shell script and manually queried against the NCBI non-redundant (nr) protein sequence database, and the results were visualized (Fig. 1D). In alignment with existing literature on immunomodulation, we retained genes with potential immunomodulatory roles for further analysis. These genes were subjected to MDS and phylogenetic tree estimation to validate their genetic and proteomic distances. Positional information of poly-GroP WTA biosynthesis-related genes was retrieved from Prokka. The *TagD-TagF1-TagF2* locus and other related genes were visualized on their respective contigs using gggenes (69).

### Mouse model validation for potential strains

The potential strain was selected based on ELISA measurements and the comparative genomic analysis results, and the PBS suspension was prepared following the same protocol as previously described with modifications, as detailed below. The pellet was weighed and adjusted to 50 mg for the preparation of the PBS suspension. The corresponding volume was transferred to a sterilized tube and heat-treated in an autoclave machine at 121°C for 20 min. For mouse model preparation:

C57BL/6 mice (10 weeks old) were fed water supplemented with or without 0.01% heat-killed LAB for 4 weeks under specific pathogen-free conditions. The spleen and Peyer’s patch cells of C57BL/6 mice were prepared as described previously (70).

### Flow cytometry

The cells were analyzed on a MACSQuant flow cytometer (Miltenyi Biotec, Tokyo, Japan) using the following specific antibodies purchased from BioLegend (San Diego, CA, USA): VioletFluo 450-labeled anti-CD19 antibodies (clone; 1D3, TONBO biosciences, San Diego, CA, USA) and Alexa647-labeled anti-GL7 antibodies (clone; GL-7), Brilliant Violet 510 anti-mouse CD4 antibodies (clone; RM4-5) and phycoerythrin (PE)-labeled anti-CD138 antibodies (clone; 281-2). Data analysis was conducted with FlowJo (FLOWJO, LLC, Ashland, OR, USA).

### Statistical analysis

The experimental data are presented as the means ± standard deviations. Statistical significance was evaluated for unpaired data using the one-way analysis of variance adjusted using Bonferroni correction.

## Data Availability

The genome data are deposited with the accession number PRJNA1146960. The accession numbers of reference genomes from NCBI for taxonomy classification are as follows. The used *L. plantarum* strains are JCM 1149 (PRJNA31515), ST-III (PRJNA52949), ZJ316 (PRJNA186807), JDM1 (PRJNA32969), WCSF1 (PRJNA356), 16 (PRJNA198762), LP91 (PRJNA221362), WJL (PRJNA294130), and 80 (PRJEB5195). The used *L. pentosus* strains are KCA1 (PRJNA81575), DSM 20314/JCM 1558 (PRJNA494616), P2000 (PRJNA896723), L33 (PRJNA729896), 9D3 (PRJNA739022), KW1 (PRJNA850900), LB-1 (PRJNA990461), and MP-10 (PRJEB14340).

## SUPPLEMENTAL MATERIAL

Supplemental material msystems.01575-24-s0001.pdf Figures S1 to S10; Tables S1 to S5 and S10 to S13.

Table S6 msystems.01575-24-s0002.xlsx *L. plantarum* top 2% orthologous gene groups screened from IL-10 comparison.

Table S7 msystems.01575-24-s0003.xlsx *L. plantarum* top 2% orthologous gene groups screened from IL-12 comparison.

Table S8 msystems.01575-24-s0004.xlsx *L. pentosus* top 2% orthologous gene groups screened from IL-10 comparison.

Table S9 msystems.01575-24-s0005.xlsx *L. pentosus* top 2% orthologous gene groups screened from IL-12 comparison.

ASM does not own the copyrights to Supplemental Material that may be linked to, or accessed through, an article. The authors have granted ASM a non-exclusive, world-wide license to publish the Supplemental Material files. Please contact the corresponding author directly for reuse.
