# Supplementary material for: Comparative genome analysis of the immunomodulatory ability of Lactiplantibacillus plantarum and Lactiplantibacillus pentosus from Japanese pickles
Source: mSystems. 2025 Apr 29;10(5):e01575-24. doi: 10.1128/msystems.01575-24 (PMC12090711; doi:10.1128/msystems.01575-24)
Supplement: Supplemental material — Figures S1 to S10; Tables S1 to S5 and S10 to S13. [file msystems.01575-24-s0001.pdf]

Comparative genome analysis of the immunomodulatory ability of *Lactiplantibacillus plantarum* and *Lactiplantibacillus pentosus* from Japanese pickles

Yiting Liu[1], Kazunori Sawada[5], Takahiko Adachi[7], Yuta Kino[1], Tingyu Yin[1], Naoyuki Yamamoto[1,6], Takuji Yamada[1,2,3,4]#

1. School of Life Science and Technology, Institute of Science Tokyo, Tokyo, Japan
2. Metagen, Inc., Yamagata, Japan
3. Metagen Theurapeutics, Inc., Yamagata, Japan
4. Digzyme, Inc., Tokyo, Japan
5. Innovation Division, Gurunavi, Inc., Hibiya Mitsui Tower, 1-1-2 Yurakucho, Chiyoda-ku, Tokyo, 100-0006, Japan
6. Laboratory for Intestinal Microbiota, Juntendo University
7. Department of Precision Health, Medical Research Institute, The Institute of Medical Engineering, Institute of New Industry Incubation, Institute of Science Tokyo, Tokyo, Japan

Address correspondence to Takuji Yamada, takuji@bio.titech.ac.jp

Fig. S1. Taxonomy identification for two species. Strains' name in pink are local *L. plantarum* strains, and strains' name in green are local *L. pentosus* strain used in this study. Highly supported nodes are indicated with a closed circle and actual value (bootstrap support value $\geq$ 50%).

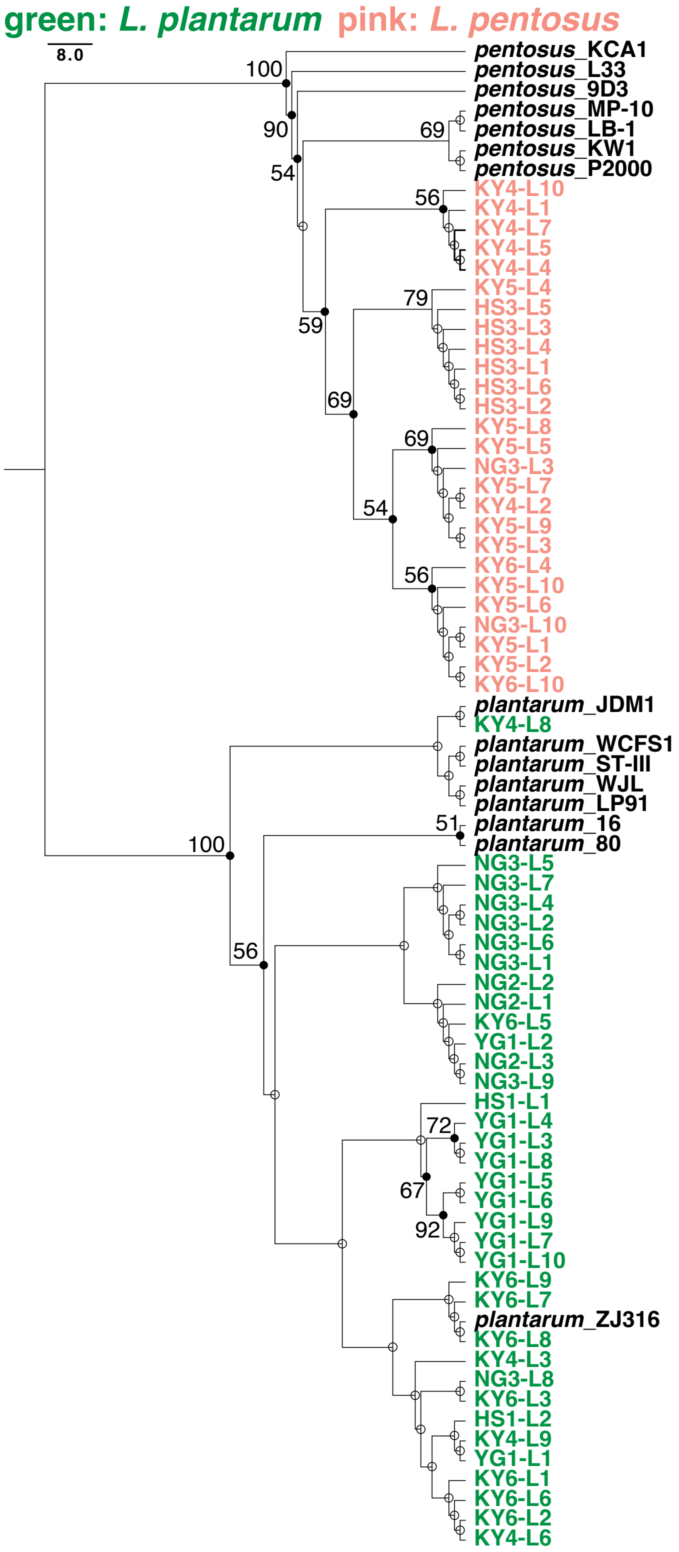

green: L. plantarum pink: L. pentosus
8.0
pentosus_KCA1
pentosus_L33
pentosus_9D3
pentosus_MP-10
pentosus_LB-1
pentosus_KW1
pentosus_P2000
KY4-L10
KY4-L1
KY4-L7
KY4-L5
KY4-L4
KY5-L4
HS3-L5
HS3-L3
HS3-L4
HS3-L1
HS3-L6
HS3-L2
KY5-L8
KY5-L5
NG3-L3
KY5-L7
KY4-L2
KY5-L9
KY5-L3
KY6-L4
KY5-L10
KY5-L6
NG3-L10
KY5-L1
KY5-L2
KY6-L10
plantarum_JDM1
KY4-L8
plantarum_WCFS1
plantarum_ST-III
plantarum_WJL
plantarum_LP91
plantarum_16
plantarum_80
NG3-L5
NG3-L7
NG3-L4
NG3-L2
NG3-L6
NG3-L1
NG2-L2
NG2-L1
KY6-L5
YG1-L2
NG2-L3
NG3-L9
HS1-L1
YG1-L4
YG1-L3
YG1-L8
YG1-L5
YG1-L6
YG1-L9
YG1-L7
YG1-L10
KY6-L9
KY6-L7
plantarum_ZJ316
KY6-L8
KY4-L3
NG3-L8
KY6-L3
HS1-L2
KY4-L9
YG1-L1
KY6-L1
KY6-L6
KY6-L2
KY4-L6

Fig. S2. Pairwise average nucleotide identity (ANI) and tetranucleotide frequency (TETRA). (A) and (C) ANI value computed for two species; (B) and (D) TETRA value computed for two species.

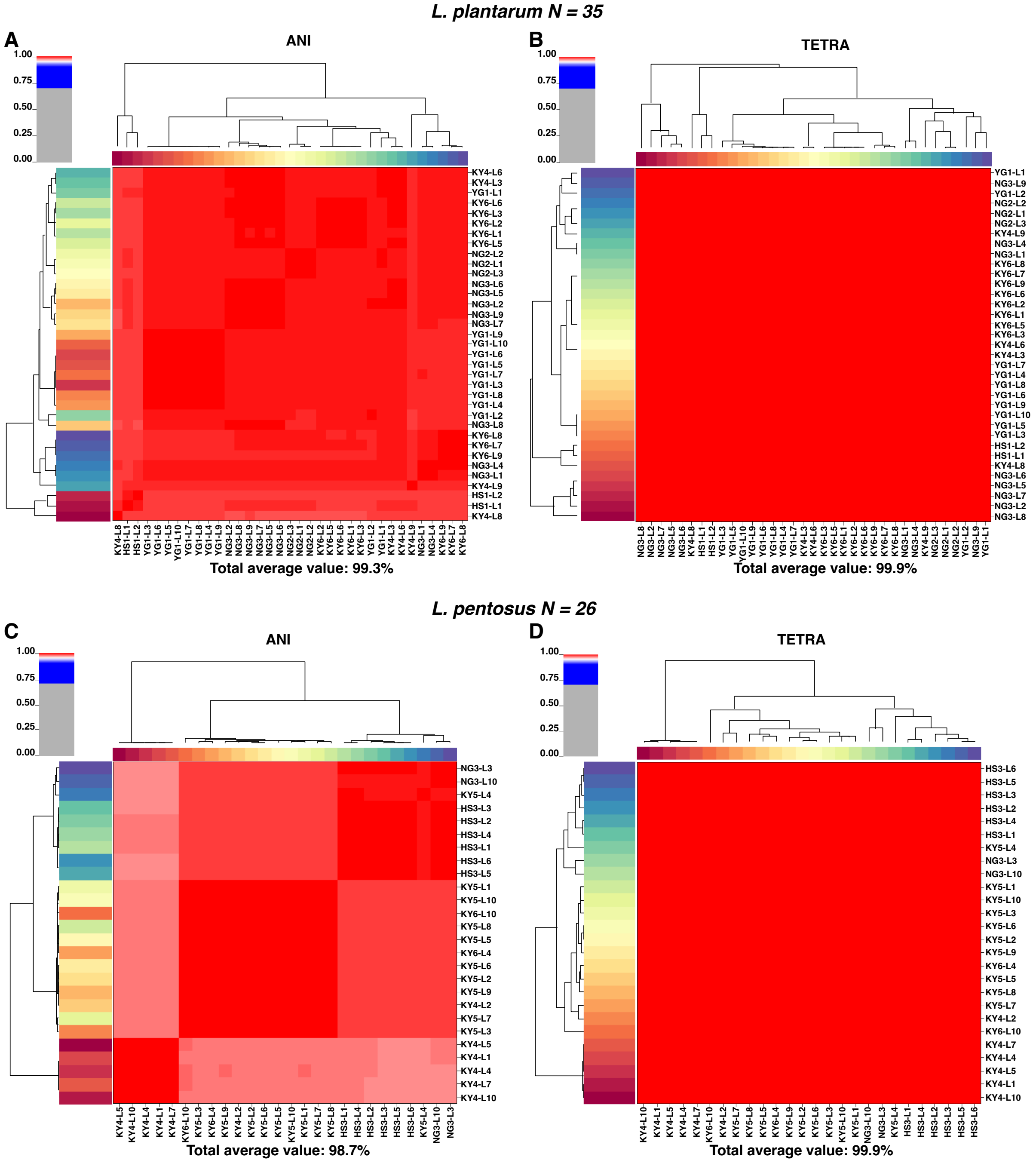

Fig. S3. Genomic statistics of two species. (A) *L. plantarum*; (B) *L. pentosus*.

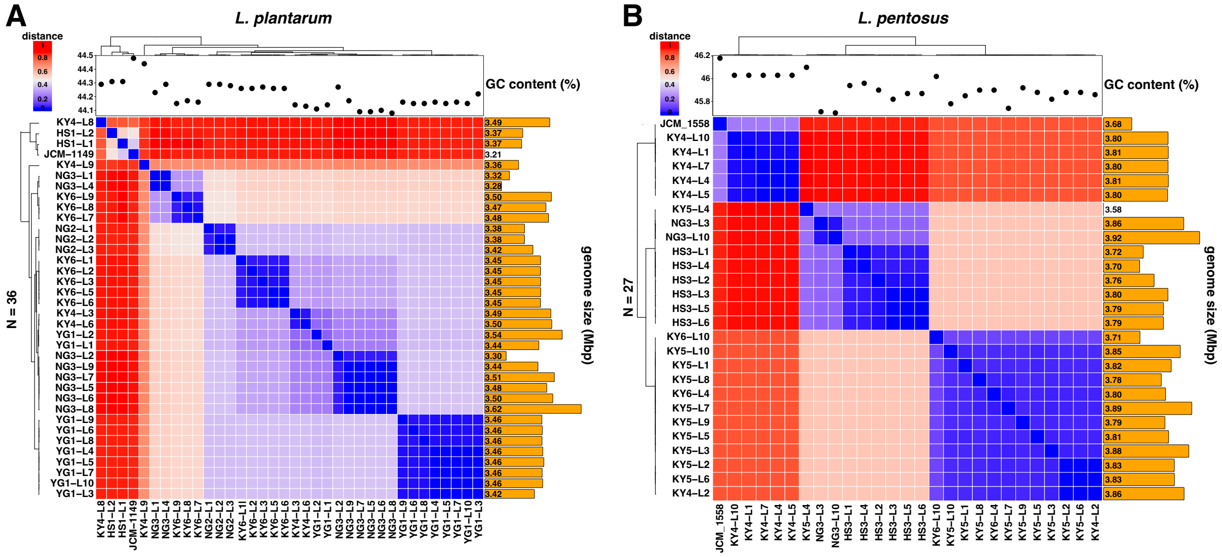

Fig. S4. Orthologous gene group composition varied across strains. (A) *L. plantarum*. (B) *L. pentosus*.

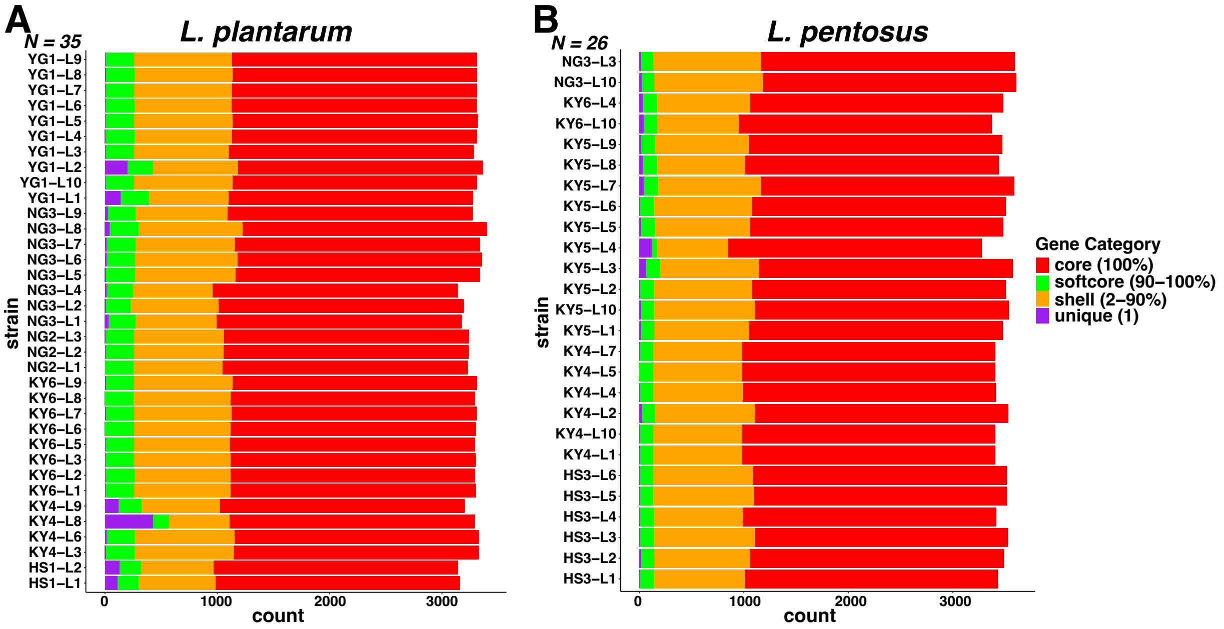

Fig. S5. Genetic distance analysis of sub-potential immunomodulation-related genes. (A) and (B) are genetic distance of *group_3159* shown in heatmaps and MDS analysis plot (n = 8); (D) and (E) are genetic distance of *group_511* shown in heatmaps and MDS analysis plot (n = 8). (C) and (F) are phylogenetic tree of *group_3159* and *group_511* based on their AA sequences, respectively. The number of strains that harbour the correspondent potential genes were indicated in ‘n’. Highly supported nodes are indicated with a closed circle and actual value (bootstrap support value ≥ 50%). Red point marked the IL-10 stimulation active strain, and blue point marked the IL-12 stimulation active strain. Clusters were shown in red or blue color to represent IL-10 or IL-12 activity.

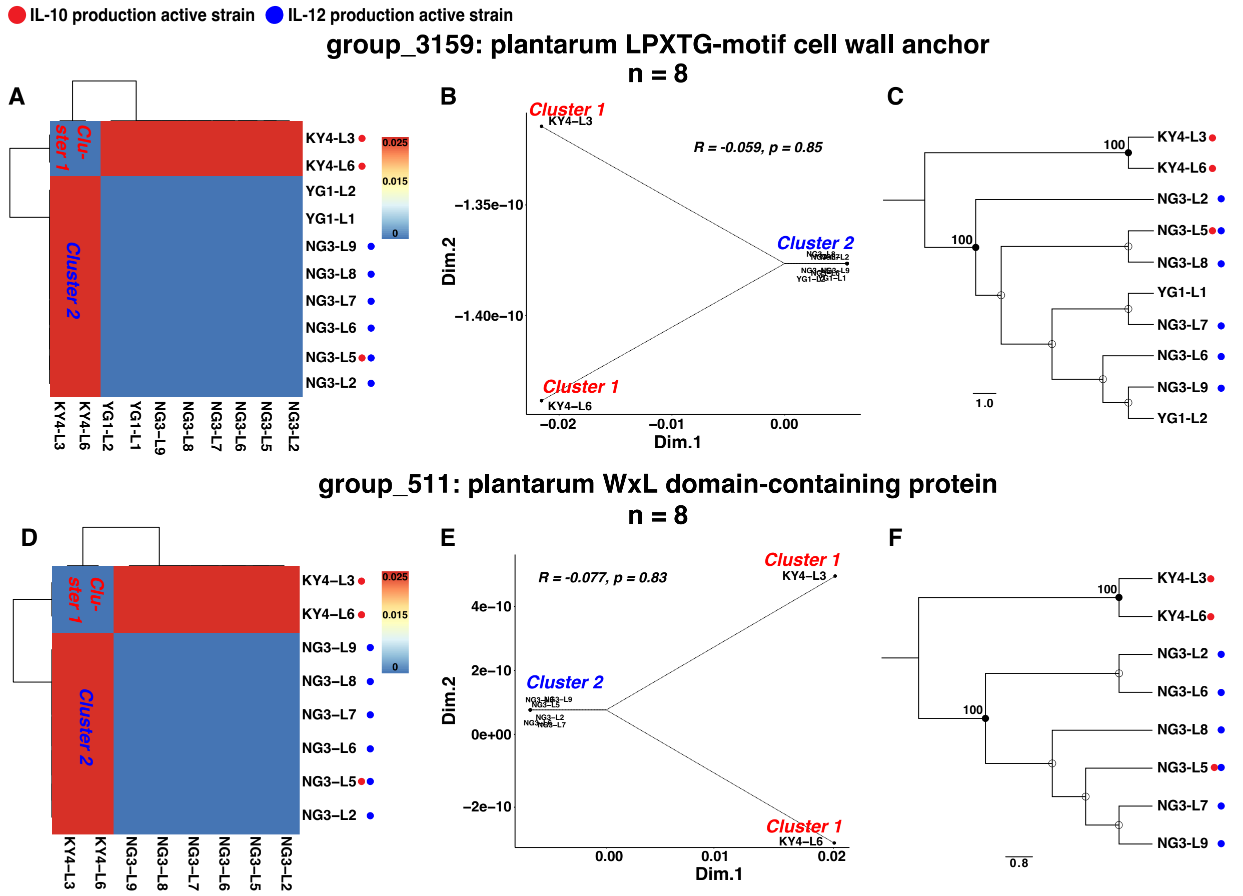

Fig. S6. *TagD-TagF1-TagF2* locus conserved by *group_1590*-possessing strains in cluster 2 (A) and cluster 1 (B) of *L. plantarum*.

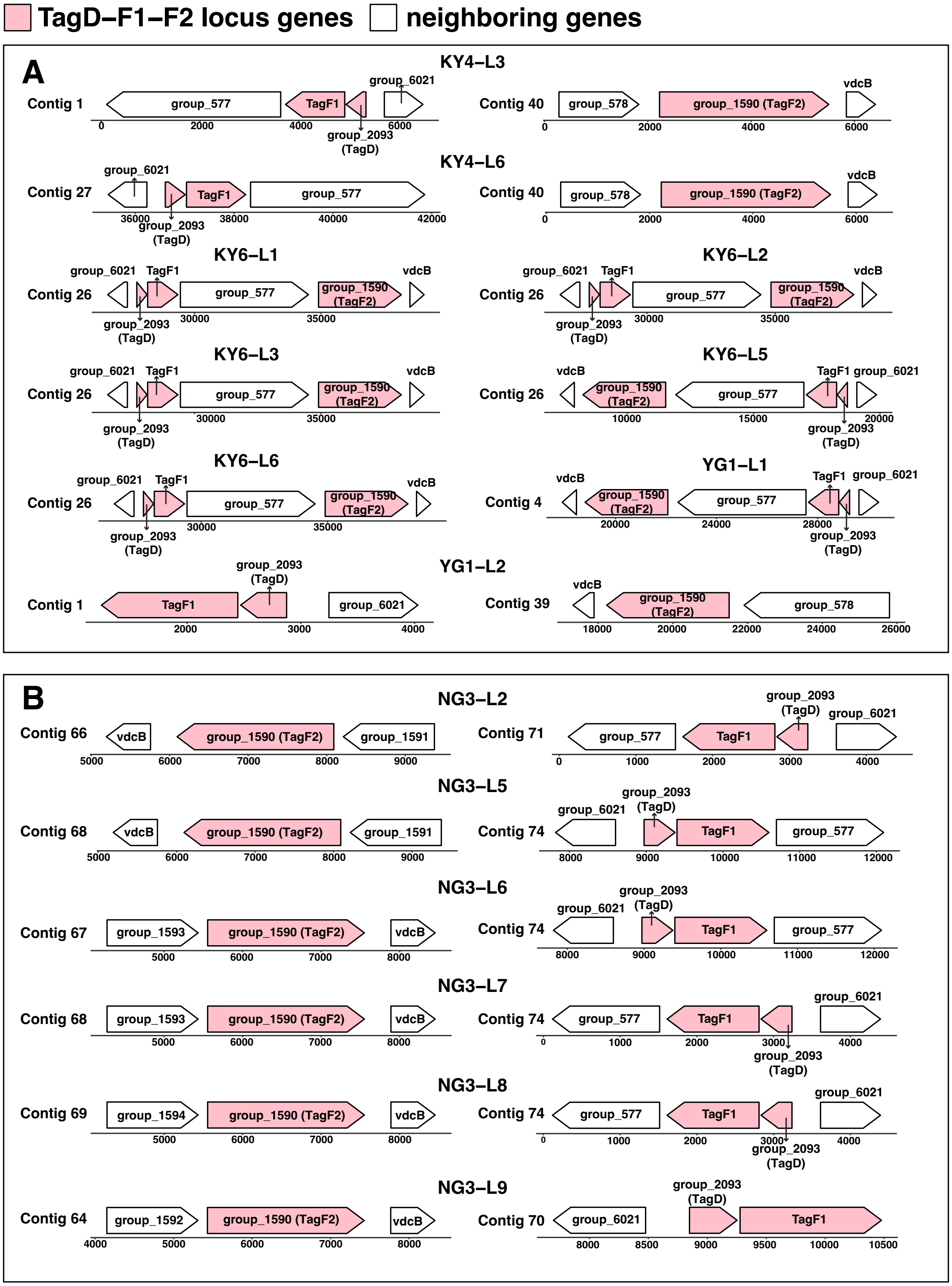
TagD–F1–F2 locus genes
neighboring genes
A
KY4–L3
Contig 1
group_577
TagF1
group_6021
group_2093
(TagD)
Contig 40
group_578
group_1590 (TagF2)
vdcB
KY4–L6
Contig 27
Contig 40
KY6–L1
KY6–L2
KY6–L3
KY6–L5
KY6–L6
YG1–L1
YG1–L2
Contig 26
Contig 4
Contig 39
B
NG3–L2
Contig 66
Contig 71
group_1591
NG3–L5
Contig 68
Contig 74
NG3–L6
Contig 67
group_1593
NG3–L7
NG3–L8
Contig 69
group_1594
NG3–L9
Contig 64
group_1592
Contig 70

Fig. S7. *TagD-TagF1-TagF2* locus conserved by *group_728*-possessing strains in cluster 2 (A) and cluster 1 (B) of *L. pentosus*.

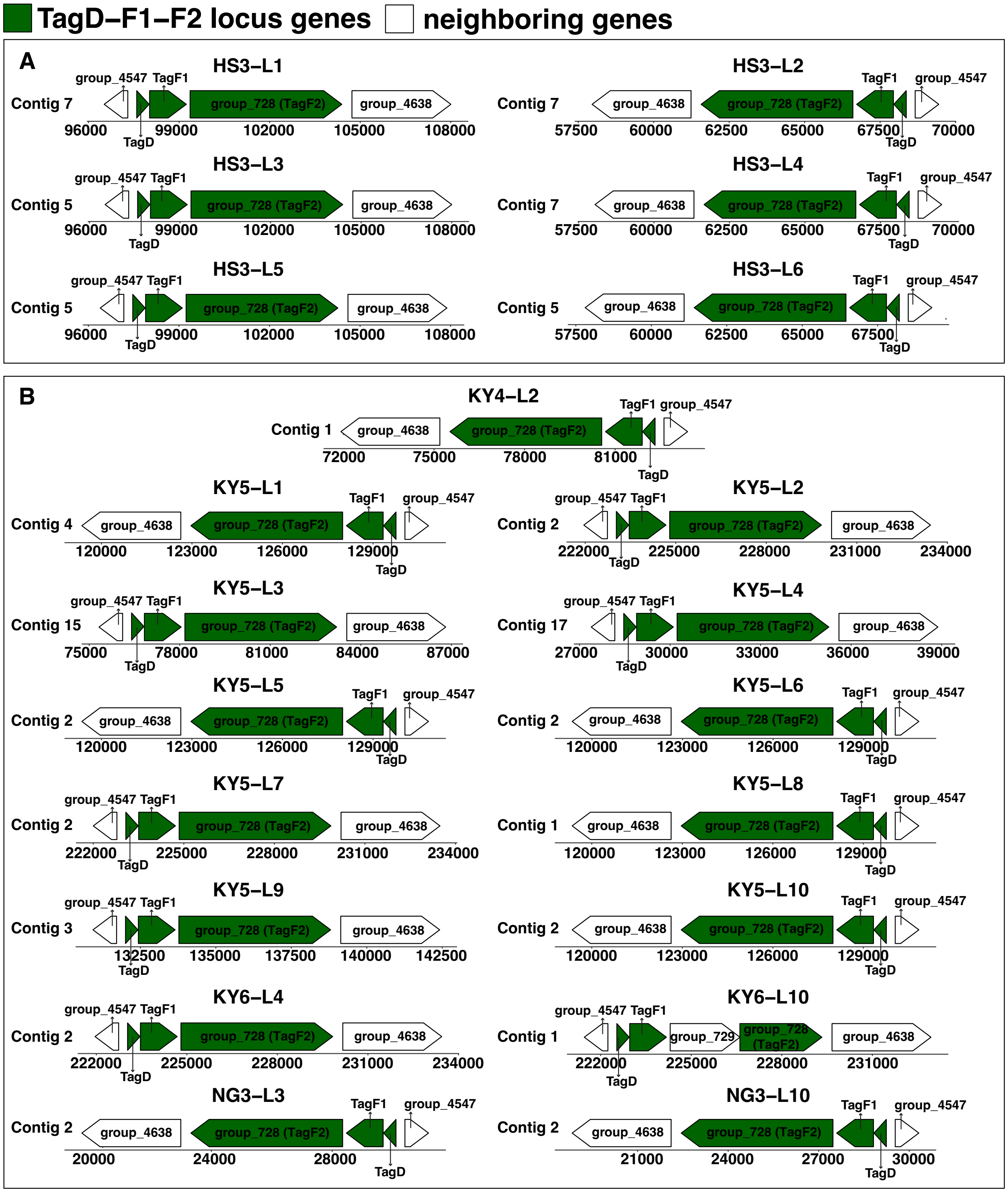

Fig. S8. The rest genes involved in poly (grop) WTA biosynthesis conserved by group_1590-possessing strains of *L. plantarum*.

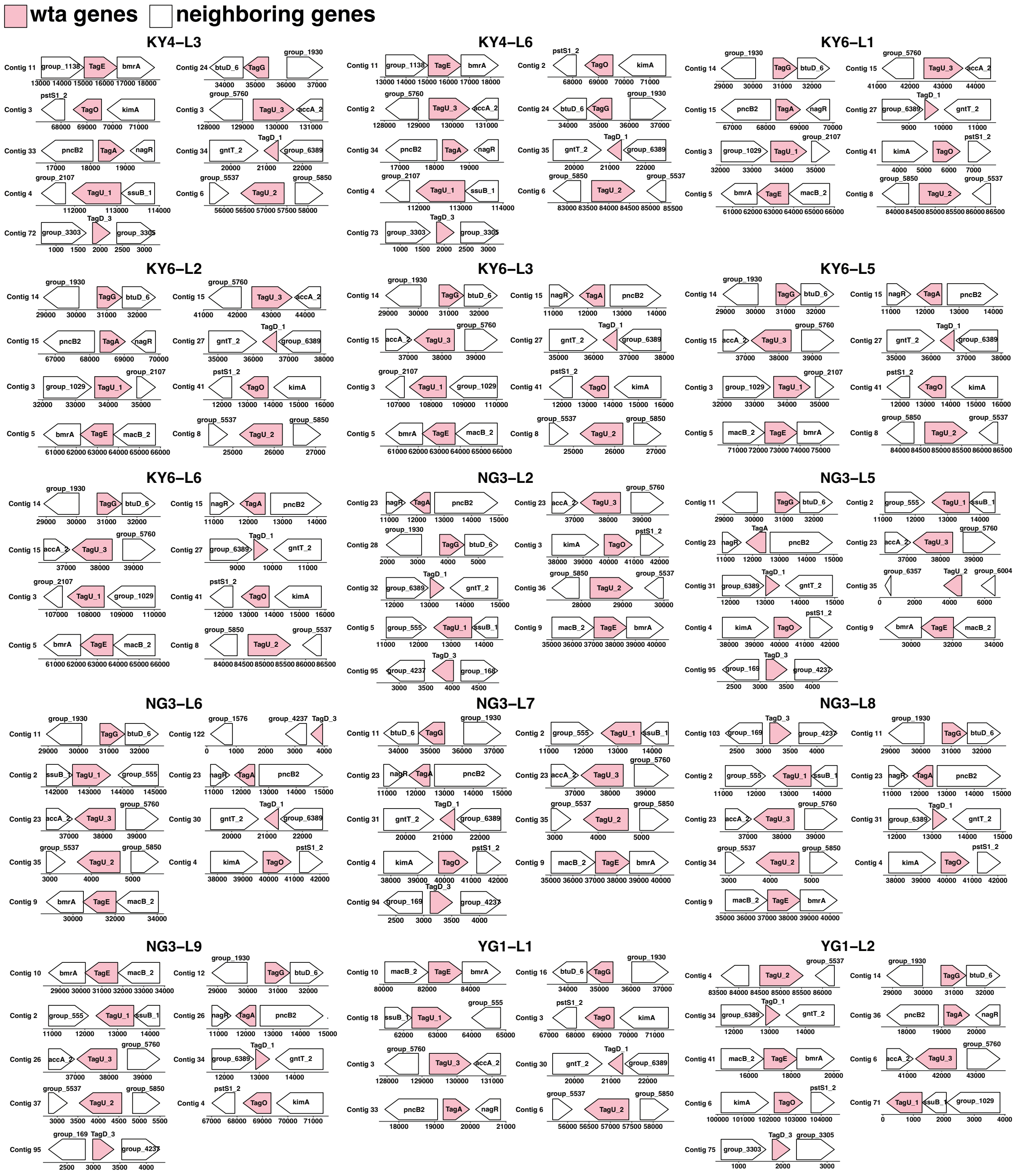

Fig. S9. The rest genes involved in poly (grop) WTA biosynthesis conserved by group_728-possessing strains of *L. pentosus*.

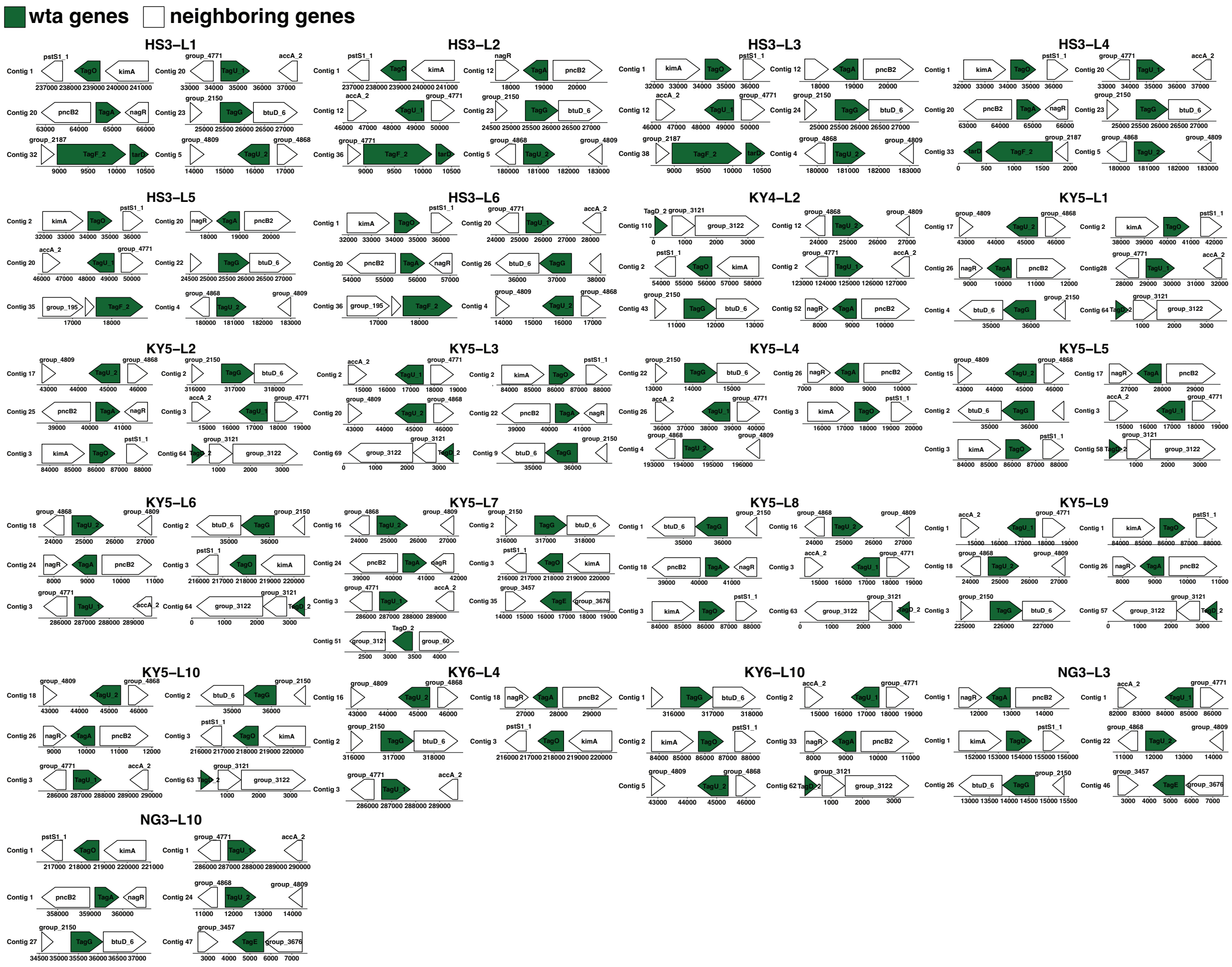

Fig. S10. Threshold-based filtration of OGs across two species. (A) IL-10-inducing comparative analysis of *L. plantarum*. (B) IL-12-inducing comparative analysis of *L. plantarum*. (C) IL-10-inducing comparative analysis of *L. pentosus*. (D) IL-12-inducing comparative analysis of *L. pentosus*. The threshold for number of active and silent strains in correspondent group was calculated as follows: n(active strains) ⩾ 80% * N (active strains) & n(silent strains) ⩽ 50% * N (silent strains). 'N (active strains)' and 'N (silent strains)' are total number of strains in active or silent group. 'n(active strains)' and 'n(silent strains)' are number of strains in each OG.

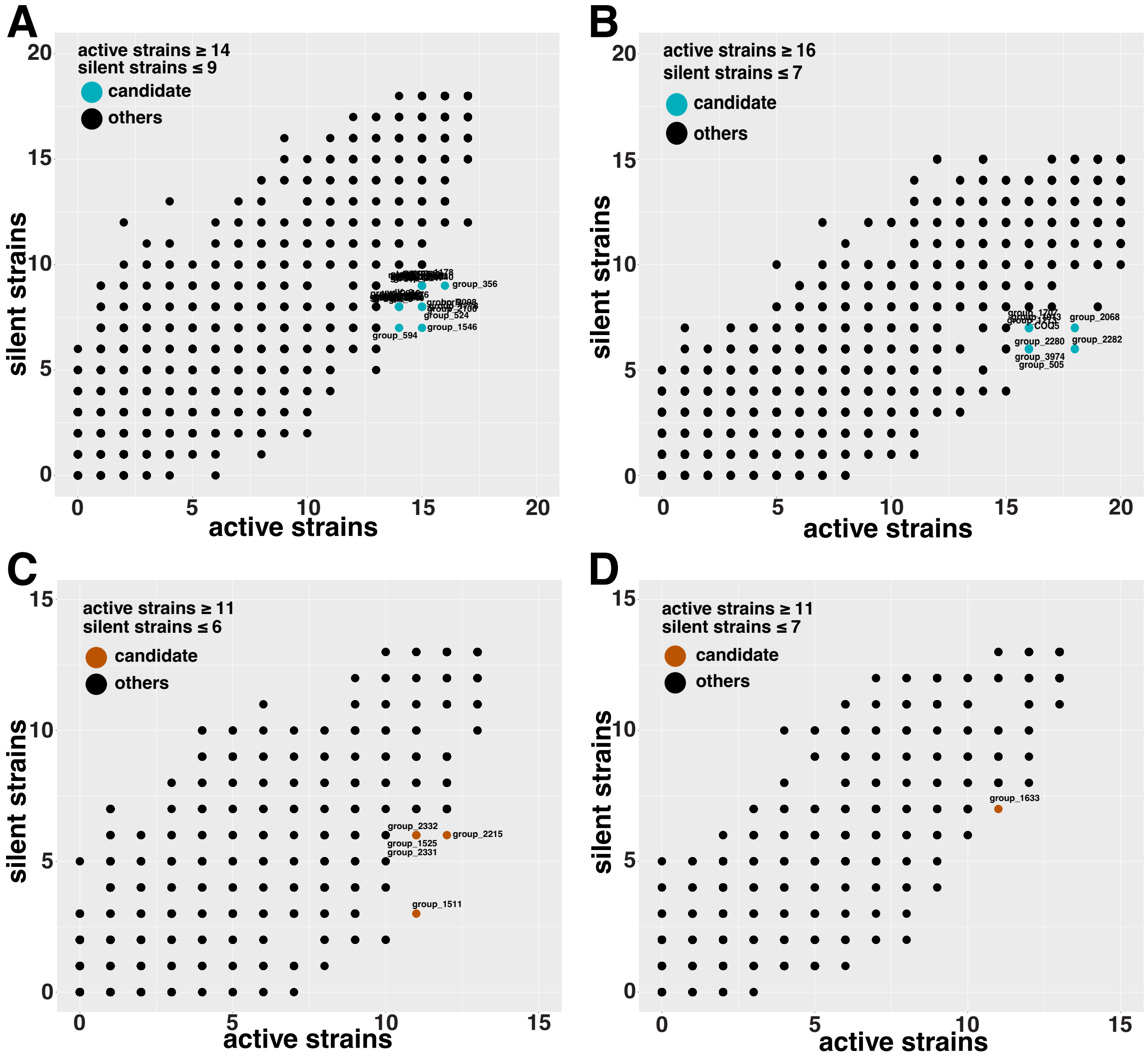

Table S1 IL-10 and IL-12 average production exhibited by active local strains across species.

	**IL-10 active strains**	**active strains avg IL-10 concn (pg/ml)**	**IL-12 active strains**	**active strains avg IL-12 concn (pg/ml)**
L. plantarum	17	7.38	20	43.43
L. pentosus	13	13.00	13	77.98

Table S2. *L. plantarum* IL-10 and IL-12 production exhibited by strains.

L. plantarum	**IL-10 trend**	**IL-10 concn (pg/ml)**	**IL-12 trend**	**IL-12 concn (pg/ml)**
**HS1-L1**	silent	0.00	silent	0.00
**HS1-L2**	silent	0.00	silent	0.00
**KY4-L3**	active	11.60	silent	0.00
**KY4-L6**	active	16.18	silent	0.00
**KY4-L8**	active	11.49	silent	0.00
**KY4-L9**	active	6.73	active	29.21
**KY6-L1**	silent	0.00	active	12.20
**KY6-L2**	active	4.17	silent	0.00
**KY6-L3**	active	8.06	active	5.41
**KY6-L5**	active	5.60	silent	0.00
**KY6-L6**	active	3.83	active	2.70
**KY6-L7**	active	4.59	silent	0.00
**KY6-L8**	active	4.07	active	2.75
**KY6-L9**	silent	0.00	active	71.18
**NG2-L1**	silent	0.00	silent	0.00
**NG2-L2**	silent	0.00	silent	0.00
**NG2-L3**	silent	0.00	active	21.16
**NG3-L1**	active	7.28	active	44.07
**NG3-L2**	silent	0.00	active	122.69
**NG3-L4**	silent	0.00	active	177.92
**NG3-L5**	active	4.90	active	23.11
**NG3-L6**	silent	0.00	active	37.74
**NG3-L7**	silent	0.00	active	84.51
**NG3-L8**	silent	0.00	active	171.49
**NG3-L9**	silent	0.00	active	38.71
**YG1-L1**	silent	0.00	silent	0.00
**YG1-L2**	silent	0.00	silent	0.00
**YG1-L3**	active	8.06	silent	0.00
**YG1-L4**	active	5.41	active	2.76
**YG1-L5**	active	6.81	silent	0.00
**YG1-L6**	active	4.50	active	3.68
**YG1-L7**	active	4.21	active	5.83
**YG1-L8**	silent	0.00	silent	0.00
**YG1-L9**	silent	0.00	active	5.53
**YG1-L10**	silent	0.00	active	5.83
**JCM-1149**	silent	0.00	active	3.81

Table S3. *L. pentosus* IL-10 and IL-12 production exhibited by strains.

L. pentosus	**IL-10 trend**	**IL-10 concn (pg/ml)**	**IL-12 trend**	**IL-12 concn (pg/ml)**
**HS3-L1**	active	15.67	active	38.27
**HS3-L2**	active	19.58	silent	0.00
**HS3-L3**	silent	0.00	silent	0.00
**HS3-L4**	silent	0.00	silent	0.00
**HS3-L5**	silent	0.00	silent	0.00
**HS3-L6**	active	2.42	active	5.55
**KY4-L1**	silent	0.00	active	217.79
**KY4-L2**	silent	0.00	active	187.91
**KY4-L4**	active	6.81	active	176.69
**KY4-L5**	silent	0.00	active	176.41
**KY4-L7**	silent	0.00	active	20.55
**KY4-L10**	silent	0.00	silent	0.00
**KY5-L1**	active	3.70	active	5.59
**KY5-L2**	silent	0.00	active	3.44
**KY5-L3**	silent	0.00	active	32.30
**KY5-L4**	active	13.24	silent	0.00
**KY5-L5**	active	10.95	silent	0.00
**KY5-L6**	active	22.00	silent	0.00
**KY5-L7**	active	8.48	silent	0.00
**KY5-L8**	active	12.56	silent	0.00
**KY5-L9**	active	48.11	silent	0.00
**KY5-L10**	active	3.23	silent	0.00
**KY6-L4**	active	2.34	active	24.59
**KY6-L10**	silent	0.00	active	118.78
**NG3-L3**	silent	0.00	silent	0.00
**NG3-L10**	silent	0.00	active	5.94
**JCM-1558**	silent	0.00	silent	0.00

Table S4. *L. plantarum* IL-10 and IL-12 production exhibited by prefectures.

Prefecture	strain number	IL-10 active	active strain avg IL-10 concn (pg/ml)	IL-12 active	active strain avg IL-12 concn (pg/ml)
**Hiroshima**	2	0	0.00	0	0.00
**Kyoto**	12	10	7.63	6	20.58
**Nagano**	11	2	6.09	9	80.16
**Yamagata**	10	5	5.80	5	4.73

Table S5. *L. pentosus* IL-10 and IL-12 production exhibited by prefectures.

**Prefecture**	**total number of strains**	**IL-10 active strain**	**avg IL-10 concn by active strain(pg/ml)**	**IL-12 active strain**	**avg IL-12 concn by active strain(pg/ml)**
**Hiroshima**	6	3	12.56	2	21.91
**Kyoto**	18	10	13.14	10	96.40
**Nagano**	2	0	0.00	1	5.94

Table S10. 31 OGs filtered from IL-10-inducing group comparison of *L. plantarum*. OGs annotation colored in red are probiotic marker genes.

**Gene**	**annotation**
**bcrR**	helix-turn-helix domain-containing protein
**bglA**	3_6-phospho-beta-glucosidase
**group_356**	glycosyltransferase
**group_524**	LysM-peptidoglycan-binding domain-containing protein
**group_594**	TetR/AcrR family transcriptional regulator
**group_1177**	hypothetical protein
**group_1178**	hypothetical protein
**group_1518**	hypothetical protein
**group_1546**	terminase TerL endonuclease subunit
**group_1778**	phage portal protein
**group_1781**	HK97 gp10 family phage protein
**group_1837**	YxeA family protein
**group_1840**	WxL domain-containing protein
**group_2076**	PTS cellobiose transporter subunit IIC
**group_2077**	hypothetical protein
**group_2098**	hypothetical protein
**group_2100**	P27 family phage terminase small subunit
**group_2485**	major tail protein
**group_2547**	sodium:proton antiporter
**group_2549**	TetR/AcrR family transcriptional regulator
**group_2940**	hypothetical protein
**group_2941**	4-carboxymuconolactone decarboxylase
**group_2942**	MerR family transcriptional regulator
**group_2944**	helix-turn-helix domain-containing protein
**group_3440**	DUF805 domain-containing protein
**hdfR_5**	transcriptional regulator
**lepA_2**	translation elongation factor 4
**nudC**	NAD(+) diphosphatase
**smc_8**	CAP domain-containing protein
**yknY**	ABC transporter ATP-binding protein
**yydK_3**	GntR family transcriptional regulator

Table S11. 9 OGs filtered from IL-12-inducing group comparison of *L. plantarum.* OGs annotation colored in red are probiotic marker genes.

Gene	annotation
**COQ5**	class I SAM-dependent methyltransferase, partial
**group_505**	PTS transporter subunit EIIC
**group_1013**	Gp15 family bacteriophage protein
**group_1273**	restriction endonuclease
**group_1707**	hypothetical protein
**group_2068**	phage tail domain-containing protein
**group_2280**	putative minor capsid protein
**group_2282**	ImmA/IrrE family metallo-endopeptidase
**group_3974**	hypothetical protein

Table S12. 5 OGs filtered from IL-10-inducing group comparison of *L. pentosus*. OGs annotation colored in red are probiotic marker genes.

Gene	annotation
**group_1511**	hypothetical protein
**group_1525**	nicotinate phosphoribosyltransferase
**group_2215**	type II toxin-antitoxin system Phd/YefM family antitoxin
**group_2331**	hypothetical protein
**group_2332**	hypothetical protein

Table S13. One OGs filtered from IL-12-inducing group comparison of *L. pentosus.* OGs annotation colored in red are probiotic marker genes.

Gene	annotation
**group_1633**	hypothetical protein
